# An ^111^In-labelled bis-ruthenium(ii) dipyridophenazine theranostic complex: mismatch DNA binding and selective radiotoxicity towards MMR-deficient cancer cells†
†Electronic supplementary information (ESI) available. See DOI: 10.1039/d0sc02825h


**DOI:** 10.1039/d0sc02825h

**Published:** 2020-08-10

**Authors:** Martin R. Gill, Michael G. Walker, Sarah Able, Ole Tietz, Abirami Lakshminarayanan, Rachel Anderson, Rod Chalk, Afaf H. El-Sagheer, Tom Brown, Jim A. Thomas, Katherine A. Vallis

**Affiliations:** a Oxford Institute for Radiation Oncology , Department of Oncology , University of Oxford , Oxford , UK . Email: Katherine.vallis@oncology.ox.ac.uk; b Department of Chemistry , University of Sheffield , Sheffield , UK; c Structural Genomics Consortium , University of Oxford , Oxford , UK; d Chemistry Research Laboratory , Department of Chemistry , University of Oxford , Oxford OX1 3TA , UK; e Department of Chemistry , Swansea University , Swansea , Wales , UK . Email: m.r.gill@swansea.ac.uk; f Chemistry Branch , Department of Science and Mathematics , Faculty of Petroleum and Mining Engineering , Suez University , Suez 43721 , Egypt

## Abstract

Theranostic radionuclides that emit Auger electrons (AE) can generate highly localised DNA damage and the accompanying gamma ray emission can be used for single-photon emission computed tomography (SPECT) imaging. Mismatched DNA base pairs (mismatches) are DNA lesions that are abundant in cells deficient in MMR (mismatch mediated repair) proteins. This form of genetic instability is prevalent in the MMR-deficient subset of colorectal cancers and is a potential target for AE radiotherapeutics. Herein we report the synthesis of a mismatch DNA binding bis-ruthenium(ii) dipyridophenazine (dppz) complex that can be radiolabelled with the Auger electron emitting radionuclide indium-111 (^111^In). Greater stabilisation accompanied by enhanced MLCT (metal to ligand charge-transfer) luminescence of both the bis-Ru(dppz) chelator and non-radioactive indium-loaded complex was observed in the presence of a TT mismatch-containing duplex compared to matched DNA. The radioactive construct [^111^In]In-bisRu(dppz) ([^111^In][In-**2**]^4+^) targets cell nuclei and is radiotoxic towards MMR-deficient human colorectal cancer cells showing substantially less detrimental effects in a paired cell line with restored MMR function. Additional cell line studies revealed that [^111^In][In-**2**]^4+^ is preferentially radiotoxic towards MMR-deficient colorectal cancer cells accompanied by increased DNA damage due to ^111^In decay. The biodistribution of [^111^In][In-**2**]^4+^ in live mice was demonstrated using SPECT. These results illustrate how a Ru(ii) polypyridyl complex can incorporate mismatch DNA binding and radiometal chelation in a single molecule, generating a DNA-targeting AE radiopharmaceutical that displays selective radiotoxicity towards MMR-deficient cancer cells and is compatible with whole organism SPECT imaging.

## Abstract 2

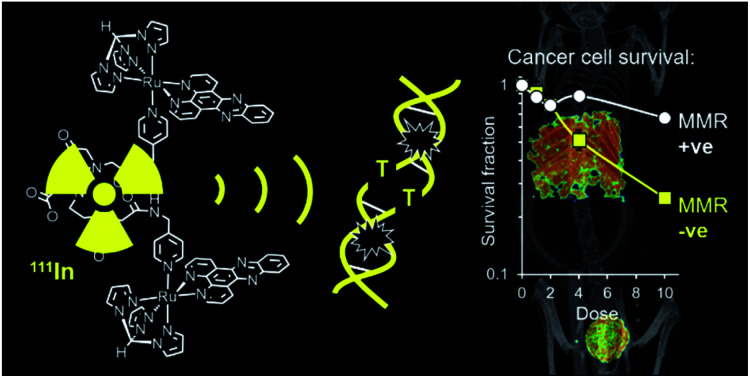
Auger electron emitter indium-111 demonstrates cancer-selective radiotoxicity and SPECT imaging compatibility when conjugated to a ruthenium(ii) polypyridyl complex.

## Introduction

The aim of targeted radionuclide therapy (TRT) is to employ a carrier molecule conjugated to a suitable radioisotope to deliver a radiotoxic dose of ionising radiation (IR) specifically to cancer cells while sparing healthy tissue.^1^,^2^ Clinical TRT radiopharmaceuticals incorporating long-range β-emitting radioisotopes most commonly target cell surface receptors or transporters.^3^ However, short-range Auger electrons (AE) emitted by radionuclides such as ^111^In (half-life = 2.8 days) are of interest as they provide high ionisation densities at the site of decay.^4^ With sufficient uptake into the cell nucleus, AEs are able to induce radiotoxic DNA damage in the form of single-strand and double-strand breaks (SSBs and DSBs), with DSBs exhibiting greater cytotoxicity.^5^ Due to the short path-length of AEs in biological media, it follows that AEs directed to specific regions within the genome will efficiently provide cell-specific radiotoxicity^6^–^8^ and that nonspecific radiotoxicity to neighbouring cells can be limited.^9^ Furthermore, many Auger electron emitting radionuclides also emit gamma ray radiation, making them compatible with whole-body SPECT imaging. This dual imaging/therapy capacity signifies AE radiotherapeutics as theranostics whereby initial diagnosis, tumour targeting and also response of the site(s) of disease to treatment may be determined by non-invasive imaging.^10^

The mismatch repair (MMR) pathway consists of a series of proteins that act to correct DNA base pair mismatches (non-Watson–Crick base pairs) generated from errors during leading- and lagging-strand replication.^11^ Direct evidence for the accumulation of mismatches has been shown in MMR-defective yeast strains^12^ and the mutation signatures in CRISPR-modified human tumour cell organoids is consistent with an approximately ×2000 greater level of mismatches in MMR-defective than MMR-proficient cells.^13^ A significant percentage of all colorectal cancers (15%) are thought to be lacking in MMR function^14^ and MMR-deficiency has been associated with resistance to common chemotherapeutics.^15^,^16^ Work utilising small molecules specifically developed for high affinity binding to mismatch sequences^17^–^20^ or employing drugs identified through repurposing screens have discovered therapeutic candidates with enhanced selectivity towards MMR-deficient human colorectal cancers.^21^–^23^ Given the success of this approach, we hypothesised that if an AE-emitting radionuclide could be targeted to a mismatch site, the resultant DNA damage would result in enhanced radiotoxicity in MMR-deficient colorectal cancer cells.

Ruthenium(ii) polypyridyl complexes (RPCs) containing intercalating ligands such as dppz (dppz = dipyrido[3,2-*a*:2′,3′-*c*]phenazine) possess high DNA binding affinities,^24^ including tunable selectivity for mismatch-containing DNA sequences,^25^–^28^ and also enhanced metal to ligand charge-transfer (MLCT) luminescence upon binding that is compatible with fluorescent cell imaging techniques.^29^–^31^ In our ongoing studies into the biological activity of RPCs, we have found Ru(ii) metallointercalators can inhibit DNA replication in cancer cells^32^,^33^ while also functioning as radiosensitizers for IR, including alongside AE radiopharmaceuticals.^34^ In particular, suitably tethered oligonuclear Ru(dppz) complexes can display high DNA binding affinities accompanied by enhanced cell uptake relative to mononuclear analogues.^35^,^36^ Consequently, we set out to use this architecture as a novel class of radiometal chelator to deliver an AE-emitting radionuclide to cellular DNA. Here, we report a bis-Ru(dppz) DNA-binding RPC designed for selectivity towards mismatch DNA and the ability to be radiolabelled with the AE-emitting radiometal ^111^In. We show this experimental radiopharmaceutical exhibits preferential radiotoxicity towards MMR-deficient cancer cells and also is suitable for use in the nuclear medicine imaging technique of SPECT in live organisms.

## Results

### Chemistry and radiolabelling

**Scheme 1:**
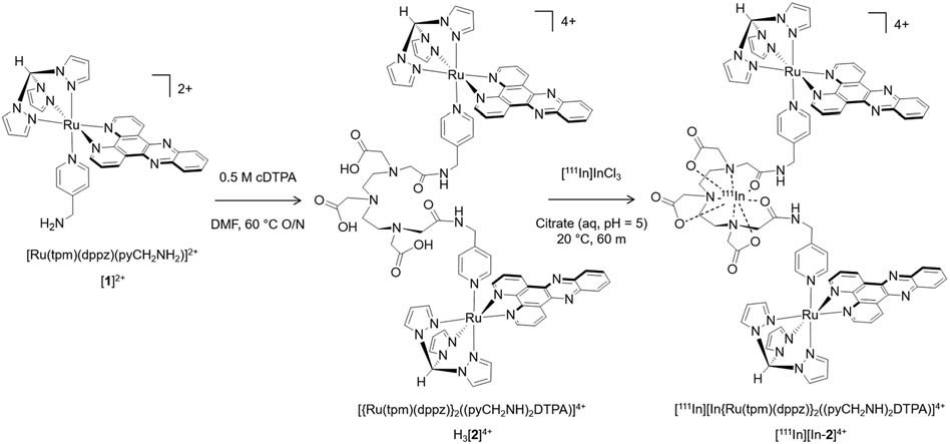
Preparation of bisRu(dppz)–DTPA chelator H_3_[**2**]^4+^ and subsequent radiolabelling with the Auger electron emitting radionuclide ^111^In to form [^111^In][In-**2**]^4+^.

The monometallic precursor [Ru(tpm)(dppz)(4-(aminomethyl)pyridine)]^2+^ (tpm = tris-(1-pyrazolyl)methane), [**1**]^2+^, was prepared from an established pathway^37^ and reacted with cDTPA (cyclic diethylenetriaminepentaacetic acid anhydride). The crude product was analysed by reverse-phase HPLC and the largest peak collected, which was determined to be the dinuclear complex H_3_[**2**]^4+^ (Scheme 1). [**1**]^2+^ and H_3_[**2**]^4+^ were analyzed by HPLC and characterised by high resolution mass spectrometry and NMR (Fig. S1 and S2 in the ESI†). As [**1**]^2+^ contains an achiral Ru(ii) centre, the structure of H_3_[**2**]^4+^ is two Ru(ii) centres each containing the intercalating ligand dppz axial to the (aminomethyl)pyridine linker. The ^111^In chelator DTPA^38^ is then at the centre of the 4-(aminomethyl)pyridine-based linker ligand between each Ru(dppz) group.

**Fig. 1:**
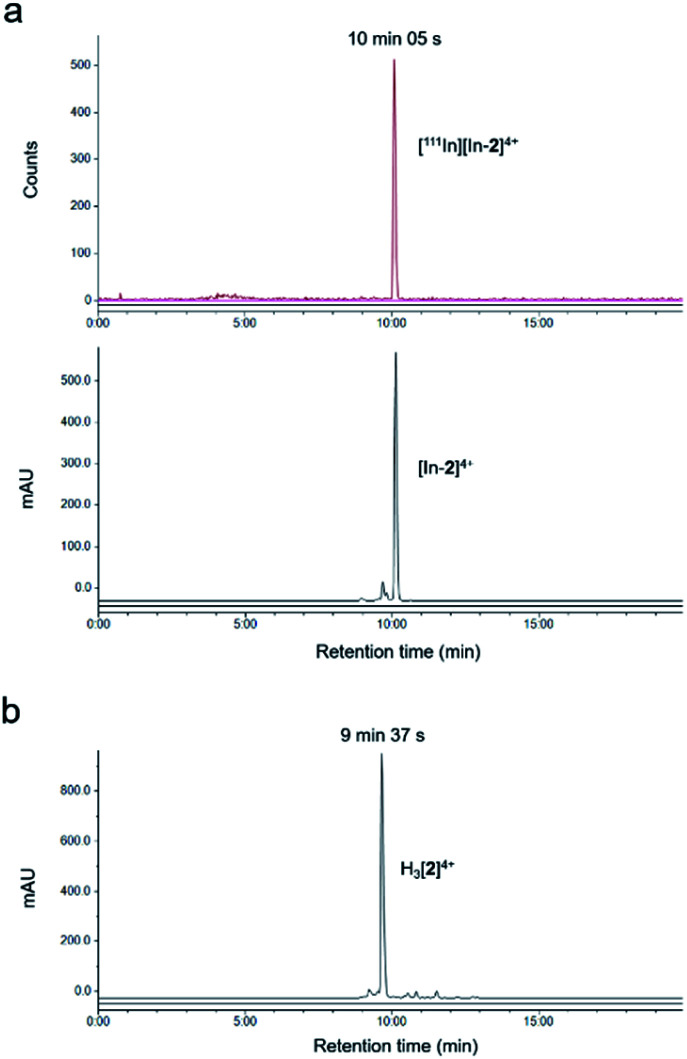
(a) HPLC chromatograms of co-injection of [^111^In][In-**2**]^4+^ and [In-**2**]^4+^ showing radioactivity (top) and absorbance (350 nm, bottom). (b) HPLC chromatogram of H_3_[**2**]^4+^ (absorbance at 350 nm). Chromatograms obtained using HPLC method B.

The non-radioactive (“cold”) In(iii)-coordinated complex, [In-**2**]^4+^, was prepared by reaction of H_3_[**2**]^4+^ with InCl_3_. Successful indium conjugation and purity of [In-**2**]^4+^ was confirmed by high resolution mass spectrometry and HPLC (Fig. S3†). Radiolabelling of H_3_[**2**]^4+^ with ^111^In was efficient under mild conditions, as determined by instant thin layer chromatography (iTLC) (Fig. S4†). HPLC analysis of [^111^In][In-**2**]^4+^ demonstrated radiochemical purity of >98% and co-injection with [In-**2**]^4+^ confirmed the identity of the radioactive species as [^111^In][In-**2**]^4+^ (Fig. 1). Specific activities of 10 MBq μg^–1^ (apparent specific molar activities of >20 GBq μmol^–1^) were achieved routinely. [^111^In][In-**2**]^4+^ was stable in aqueous solution at room temperature, with no ^111^In dissociation for up to 72 h storage (Fig. S5†). No significant dissociation of ^111^In^3+^ from [^111^In][In-**2**]^4+^ in cell media at 37 °C, in the presence of serum, or competing metal ions Fe^3+^ or Zn^2+^ was observed (Fig. S6†).

### DNA binding and TT mismatch selectivity

**Fig. 2:**
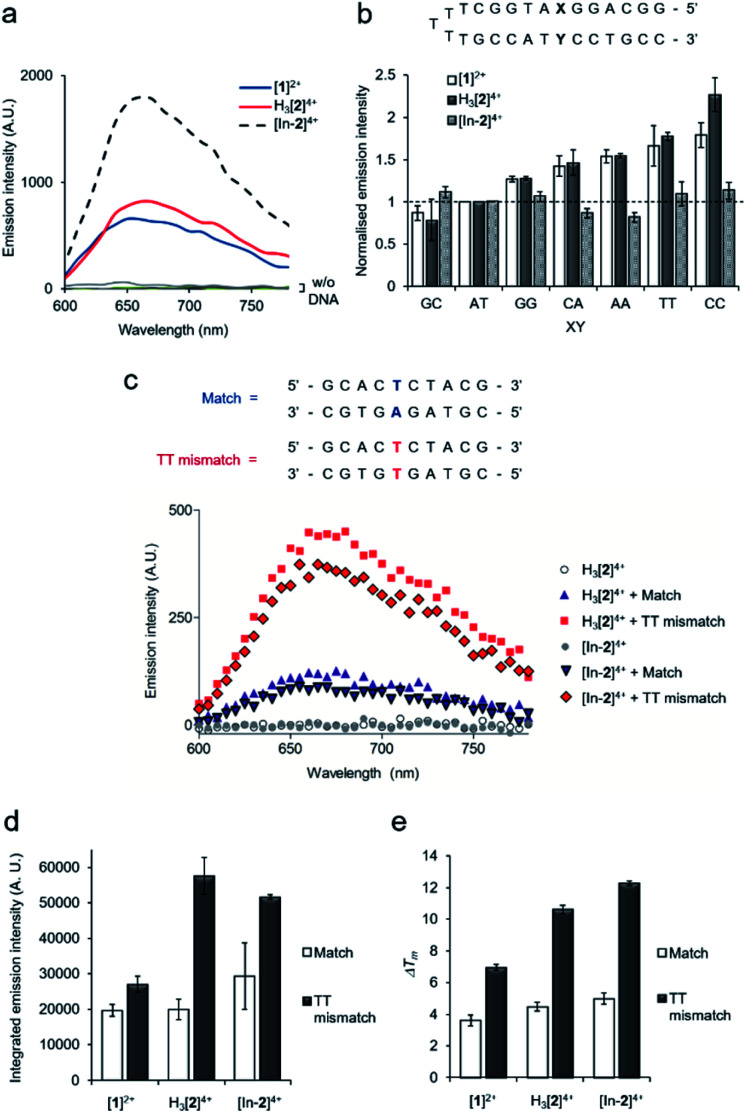
(a) MLCT luminescence of Ru(ii) complexes (30 μM) without (w/o) and with calf-thymus DNA (80 μM) (*λ*_ex_ = 405 nm). (b) Relative emission intensities for [**1**]^2+^, H_3_[**2**]^4+^ or [In-**2**]^4+^ (30 μM) with the addition of DNA hairpins containing a single variable XY base pair (3 μM, top). Data for each compound normalised to XY = AT emission intensity (dashed line). Mean of two independent replicates ± S.D. (c) Emission spectra of H_3_[**2**]^4+^ or [In-**2**]^4+^ with 10-mer matched DNA duplex or 10-mer containing a single TT mismatch site (duplex sequences shown at top, 30 μM complex, 3 μM DNA duplex). (d) Integrated emission intensities TT mismatch and matched DNA with the addition of [**1**]^2+^, H_3_[**2**]^4+^ or [In-**2**]^4+^ (3 μM DNA duplexes, 30 μM complexes). Average of two independent experiments ± S.D. (e) Δ*T*_m_ (°C) values for match or TT mismatch duplexes (3 μM) with the addition of complexes (30 μM), determined by thermal denaturation studies (each *T*_m_ average of six successive melting curves per condition). *T*_m_ (match) = 47.43 °C, *T*_m_ (TT mismatch) = 33.62 °C. Error bars represent the combined standard uncertainty. Conditions: 10 mM phosphate buffer, 200 mM NaCl, pH 7.0. *λ*_ex_ = 405 nm, *λ*_em_ = 600–800 nm for [**1**]^2+^, H_3_[**2**]^4+^ and [In-**2**]^4+^.

All complexes demonstrated a distinctive increase of MLCT emission upon addition of CT-DNA (calf thymus DNA), indicative of DNA intercalation of the dppz moiety,^29^ with [In-**2**]^4+^ displaying the greatest emission enhancement (Fig. 2a). DNA binding constants, *K*_b_, were obtained by fitting binding curves from luminescence DNA titrations to the McGhee von Hippel binding model (Fig. S7 and Table S1†).^39^ The trimetallic species [In-**2**]^4+^ (*K*_b_ = 1.1 × 10^6^ M^–1^) demonstrated greater affinity for CT-DNA than the mononuclear complex [**1**]^2+^ (*K*_b_ = 3.8 × 10^5^ M^–1^), unsurprising as [In-**2**]^4+^ contains two Ru(dppz) groups and a greater overall positive charge. The native H_3_[**2**]^4+^ (*K*_b_ = 2.4 × 10^5^ M^–1^) chelator showed a lower binding affinity than [In-**2**]^4+^, indicating that indium-loading acts to increase DNA affinity. As H_3_[**2**]^4+^ will exist as the monocationic [**2**]^+^ species in aqueous solution at pH 7 due to deprotonation of the three hydroxyl groups of the DTPA linker, this may be rationalised by the greater positive charge of [In-**2**]^4+^.

Next, selectivity for mismatched DNA was examined. Other work has shown that increasing the steric demand of ancillary ligands attached to the Ru(dppz) results in complexes that preferentially bind to mismatches.^25^–^28^ We reasoned that [**1**]^2+^ and H_3_[**2**]^4+^ may show similar selectivity as each possess the bulky tpm ancillary ligand and a Ru(dppz) centre(s). Employing a DNA hairpin containing a single variable base pair, a greater relative emission for [**1**]^2+^ or H_3_[**2**]^4+^ for every hairpin containing a mismatch base pair (GG, CA, AA, TT and CC) compared to a well-matched AT or GC base pair was seen (Fig. 2b). Although, on initial inspection, these results appear similar to work employing the related DNA mismatch “light switch” molecule [Ru(Me_4_phen)_2_(dppz)]^2+^ (Me_4_phen = 3,4,7,8-tetramethyl-1,10-phenanthroline),^27^ a significant increase for the TT mismatch-containing sequence was apparent for H_3_[**2**]^4+^. This behavior was not observed for [Ru(Me_4_phen)_2_(dppz)]^2+^ and is a relatively rare finding for mismatch-interactive compounds.^20^

The emissive properties of the compounds towards a 10-mer oligomeric DNA duplex containing a single TT mismatch was examined (Fig. 2c and S8†). Compared to the well-matched sequence, H_3_[**2**]^4+^ showed an enhanced emission intensity for the TT mismatch-containing duplex (Fig. 2c and d), suggesting a stronger binding interaction due to presence of the mismatch. Thermal denaturation studies of each DNA duplex in the absence and presence of each complex were performed to further elucidate this matter. Ligand-induced changes of DNA melting temperature (Δ*T*_m_) indicate that all the Ru(ii) complexes increased the melting temperature of the matched duplex (Δ*T*_m_ values of 3.6 ± 0.3, 4.5 ± 0.3 and 5.0 ± 0.4 °C for [**1**]^2^, H_3_[**2**]^4+^ and [In-**2**]^4+^ respectively, Fig. 2e). Notably, the increase in *T*_m_ values with mismatch-containing duplexes due to incubation with each Ru(ii) complex were significantly larger than for the well-matched sequence (Δ*T*_m_ values 7.0 ± 0.2, 10.6 ± 0.3 and 12.3 ± 0.2 °C for the addition of [**1**]^2^, H_3_[**2**]^4+^ and [In-**2**]^4+^ to the mismatch-containing sequence respectively, Fig. 2e), indicating greater stabilisation of the duplex when the mismatch TT base pair is present. This increased mismatch stabilisation was more pronounced for H_3_[**2**]^4+^ and [In-**2**]^4+^ than [**1**]^2+^; an observation that is in alignment with the luminescence data. [In-**2**]^4+^ did not demonstrate mismatch selectivity with the hairpin structure (Fig. 2b), the luminescence and Δ*T*_m_ values for [In-**2**]^4+^ and H_3_[**2**]^4+^ showed comparable increases for the 10-mer duplex containing a TT mismatch over the well-matched sequence (Fig. 2c–e). This indicates that In-coordination does not interfere with preferential TT mismatch stabilisation within a duplex environment. The contrasting results obtained for the hairpin DNA likely indicates altered geometric or entropic contributions to binding as a result of In-coordination decreasing the flexibility of the molecule.

### Cellular uptake and cytotoxicity of non-radioactive complexes

**Fig. 3:**
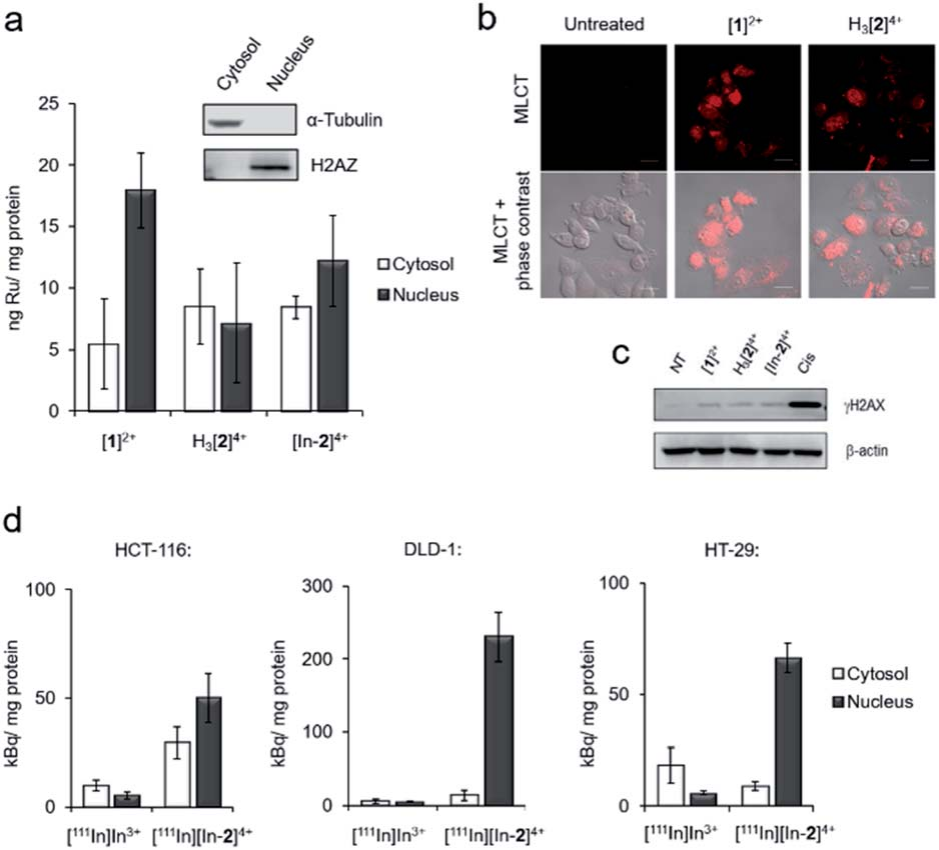
(a) Intracellular Ru content of HCT-116 cells treated with [**1**]^2+^, H_3_[**2**]^4+^ or [In-**2**]^4+^ (50 μM, 2 h), as determined by ICP-MS. Cells were separated into cytosol and nuclear fractions before analysis (see inset for verification of successful fractionation). Mean of triplicates ± S.D. (b) Confocal laser scanning micrograph of HCT-116 cells treated with [**1**]^2+^ or H_3_[**2**]^4+^ (100 μM, 4 h) showing nuclear MLCT emission. Scale bars = 20 μm. (c) Western blotting for γH2AX levels in HCT-116 cells after 24 treatment with cisplatin (Cis, 7 μM) or Ru(ii) compounds (50 μM). β-Actin was used as a loading control. NT = not treated. (d) Cytosolic and nuclear uptake of [^111^In][In-**2**]^4+^ in DLD-1, HCT-116 or HT-29 cells (1 MBq ml^–1^, 2 h), as determined by radioactivity in cytosolic and nuclear subcellular fractions. An equivalent amount of radioactivity of [^111^In]In^3+^ was included for comparative purposes. Mean of triplicates ± S.D.

Inductively coupled plasma mass spectroscopy (ICP-MS) analysis of HCT-116 human colorectal cancer cells treated with [**1**]^2+^, H_3_[**2**]^4+^ or “cold” [In-**2**]^4+^ showed relatively low cellular uptake of each complex with a significant proportion of ruthenium content detected in isolated nuclear fractions (Fig. 3a). For example, cells treated with 50 μM [**1**]^2+^ for 2 h, 5.5 ± 3.6 and 17.9 ± 3.0 ng Ru per mg cell protein was detected in the cytosol and nucleus, respectively. Although intracellular Ru content was comparable for all complexes, H_3_[**2**]^4+^ and “cold” [In-**2**]^4+^ are both dinuclear Ru complexes and so therefore have decreased uptake of each molecule compared to the mononuclear [**1**]^2+^. These results are in contrast to recent results employing a hydrophobic linker between Ru(tpm)(dppz) centres^36^ and are likely explained by the hydrophilicity of DTPA acting to decrease cellular uptake. Comparable results for H_3_[**2**]^4+^ and [In-**2**]^4+^ were seen (cytosolic Ru content: 8.5 ± 3.0 and 8.4 ± 0.9 ng Ru per mg cell protein for H_3_[**2**]^4+^ and [In-**2**]^4+^ respectively. Nuclear Ru content: 7.2 ± 4.9 and 12.2 ± 3.7 ng Ru per mg cell protein for cells treated with H_3_[**2**]^4+^ and [In-**2**]^4+^ respectively, Fig. 3a), indicating that In-coordination did not substantially alter cellular uptake or subcellular distribution of the Ru(ii) scaffold. MLCT emission detectable by confocal laser scanning microscopy (CLSM) also provides evidence of *in cellulo* DNA binding.^40^ Evidence of MLCT luminescence was observable in the nuclei of live HCT-116 cells treated with [**1**]^2+^ or H_3_[**2**]^4+^ (Fig. 3b), which does supply evidence that nuclear DNA is targeted. Intracellular luminescence was generally poor (Fig. S9†), preventing more extensive use of this technique. Low intracellular MLCT emission is likely due to the low cellular uptake of the complexes. For example, the RPC nuclear imaging agent [Ru(phen)_2_(tpphz)]^2+^ (phen = 1,10 phenanthroline, tpphz = tetrapyridophenazine) demonstrates 100-fold greater uptake than the complexes within this work.^33^ Erratic nuclear staining appears to be a common feature of Ru(dppz) complexes, even for molecules with more pronounced “light switch” effects.^24^,^30^

[**1**]^2+^ and H_3_[**2**]^4+^ demonstrated low cytotoxicity, with 72 h half-inhibitory IC_50_ concentrations >50 μM in all lines tested (Fig. S10 and Table S3†). [In-**2**]^4+^ showed mild cytotoxicity towards HCT-116 and HeLa cells, approximately five-fold less cytotoxic than cisplatin in HCT-116 cells (IC_50_ concentrations of [In-**2**]^4+^: 34 and 32 μM towards HCT-116 and HeLa cells, respectively. Cisplatin: 6.6 and 0.5 μM towards HCT-116 and HeLa cells, respectively, Table S3†). Unlike more potent anti-proliferative Ru(ii) metallointercalators,^32^,^33^ no significant enhancement of the DSB damage marker γH2AX (H2AX phosphorylated at Ser139) (ref. 41) above levels found in untreated control cells was seen in response to treatment with any Ru(ii) compound (Fig. 3c).

### Cellular uptake of [^111^In][In-**2**]^4+^

Cellular uptake of the radiolabelled complex [^111^In][In-**2**]^4+^ (log *P* = –2.46 ± 0.26) was examined in three human colorectal cancer cell lines: DLD-1 and HCT-116, which are both MMR-deficient and hypermutated, and HT-29, which is MMR proficient and nonhypermutated (Table S2† and ref. 42). These results showed greater uptake of [^111^In][In-**2**]^4+^ than the non-coordinated “free” [^111^In]In^3+^ in all cell lines tested and the greatest radioactivity was seen in the isolated nuclear fractions, indicating successful nuclear targeting by [^111^In][In-**2**]^4+^ (Fig. 3d). A comparable subcellular distribution of radioactivity to intracellular ruthenium content (by ICP-MS) in HCT-116 cells for [^111^In][In-**2**]^4+^/[In-**2**]^4+^ is consistent with ^111^In/In-coordination to H_3_[**2**]^4+^ remaining stable in cells. Interestingly, elevated nuclear uptake of [^111^In][In-**2**]^4+^ was apparent in DLD-1 cells, with approximately four-fold greater levels of radioactivity in this fraction compared to HCT-116 or HT-29 cells (Fig. 3d).

### Radiotoxicity of [^111^In][In-**2**]^4+^

**Fig. 4:**
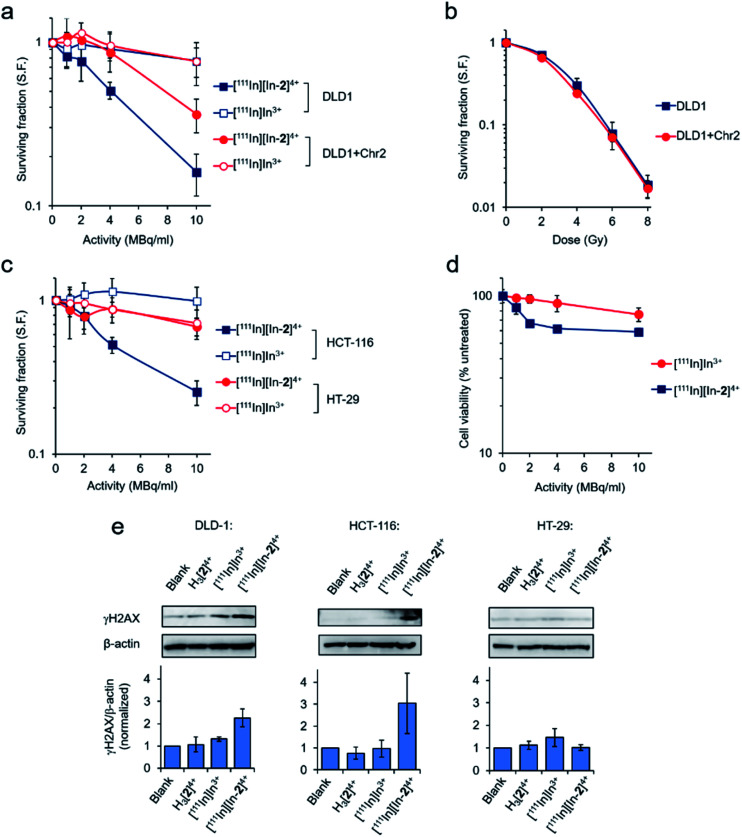
(a) Radiotoxicity of [^111^In][In-**2**]^4+^ towards DLD-1 (MMR-deficient) or DLD-1 + Chr2 (MMR restored) human colorectal cancer cell lines, as determined by clonogenic survival assay (24 h incubation time). Mean ± S.D. of three independent experiments, where each experiment was performed in triplicate. (b) Clonogenic survival of DLD-1 or DLD-1 + Chr2 cells after irradiation with ^137^Cs-γ-rays. Mean ± S.D. of three independent experiments, where each experiment was performed in triplicate. (c) Radiotoxicity of [^111^In][In-**2**]^4+^ towards MMR-deficient HCT-116 or MMR-proficient HT-29 cancer cell lines, as determined by clonogenic survival assay (24 h incubation time). Mean ± S.D. of triplicates. (d) Cell viability of WI-38 normal human fibroblasts treated with [^111^In][In-**2**]^4+^ (24 h incubation time, cell viability measured by MTT assay 5 days after complex removal). Mean ± S.D. of triplicates. (e) DNA damage in cell lines treated with [^111^In][In-**2**]^4+^ (10 MBq ml^–1^, 6 h), as determined by γH2AX levels. Mean of two technical repeats ± S.D. Specific activity of [^111^In][In-**2**]^4+^ = 20 MBq μg^–1^, corresponding to a concentration of H_3_[**2**]^4+^ < 0.5 μM in all experiments. Equivalent doses of [^111^In]In^3+^ (by activity ml^–1^, as [^111^In]In citrate) or H_3_[**2**]^4+^ (by concentration) were included for comparative purposes, where indicated.

Employing the DLD-1 and DLD-1 + Chr2 paired cell lines, where DLD-1 + Chr2 cells are DLD-1 cells with genetically restored MMR function, greater radiotoxicity of [^111^In][In-**2**]^4+^ towards the parental DLD-1 cells was apparent with reduced effects towards the MMR restored DLD-1 + Chr2 cells (Fig. 4a). Low radiotoxicity was observed for equivalent doses of free [^111^In]In^3+^ in both cell lines. MMR status in the matched cell line pair has no impact on cell survival in response to external beam γ-rays, indicating no change in inherent radiosensitivity due to the restoration of MMR function (Fig. 4b). As there was negligible cytotoxicity from the non-radioactive complex at the low concentrations of H_3_[**2**]^4+^ employed (Fig. S10 and Table S3†), these findings are consistent with the potency of [^111^In][In-**2**]^4+^ being attributable to ^111^In-induced radiotoxicity. [^111^In][In-**2**]^4+^ was similarly radiotoxic towards MMR-deficient HCT-116 cells but showed substantially reduced radiotoxicity towards the MMR-proficient HT-29 cells and normal WI-38 human fibroblasts (Fig. 4c and d). Examining levels of DNA damage in cells treated with [^111^In][In-**2**]^4+^, greater expression of γH2AX was seen in DLD-1 and HCT-116 cell lines compared to HT-29 cells (Fig. 4e). Negligible DNA damage generation as a result of treatment with [^111^In]In^3+^ or the non-radiolabelled H_3_[**2**]^4+^ (Fig. 4e) provided further evidence of the targeted radiotoxicity of [^111^In][In-**2**]^4+^. γH2AX foci formation due to [^111^In][In-**2**]^4+^ treatment was confirmed in HCT-116 cells by immunofluorescence (Fig. S11†).

Metal complexes can also function as radiosensitizers for DNA-damaging IR, either as a result of their biological activity and/or the presence of an atom with a high *Z* number.^43^ Treatment of HCT-116 cells with 50 μM H_3_[**2**]^4+^ or “cold” [In-**2**]^4+^ did not result in significant enhancement of cellular sensitivity to γ-rays (Fig. S12 and Table S4†), indicating that neither the chelator nor non-radioactive In-coordinated complex have potent radiosensitizing effects. Considering the low concentrations of H_3_[**2**]^4+^ employed in cell survival studies (<0.5 μM), a “self-radiosensitizing” effect contributing to the radiotoxicity of [^111^In][In-**2**]^4+^ is unlikely.

### SPECT imaging and biodistribution

**Fig. 5:**
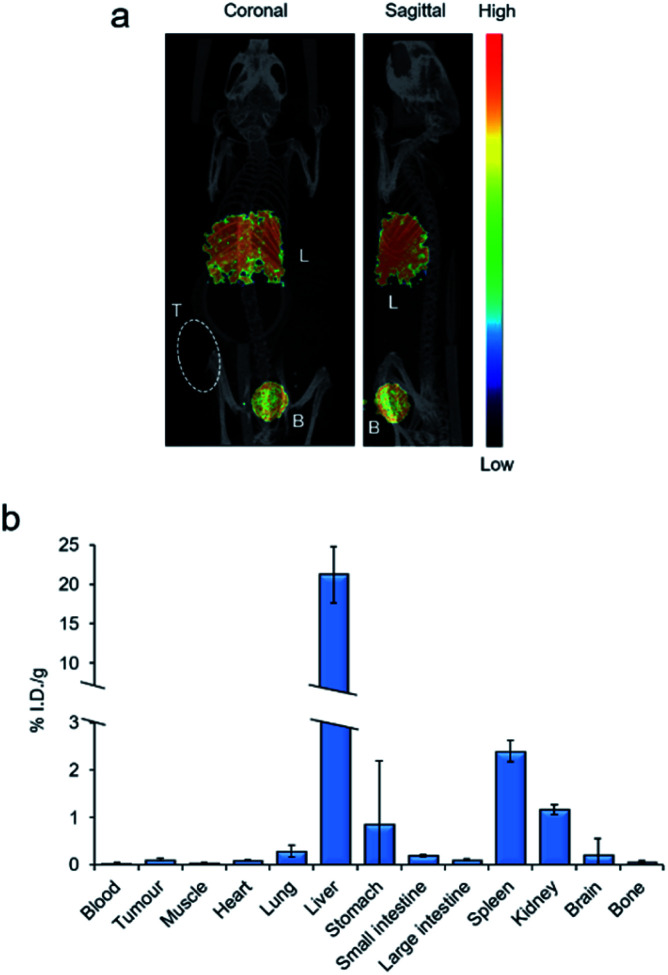
(a) Representative SPECT/CT image of a mouse constructed from images taken 10–70 min after intravenous injection of [^111^In][In-**2**]^4+^ (∼8 MBq). L = liver. B = bladder. T = DLD-1 xenograft tumour. (b) *Ex vivo* radioactivity content of organs of mice 24 h after intravenous injection of [^111^In][In-**2**]^4+^ expressed as the % of injected dose (at *t* = 0 h) per gram (*n* = 3, mean ± S.D.). Note break in *y*-axis.

In addition to AEs, ^111^In also emits γ-rays, making the radionuclide compatible with SPECT imaging, a technique commonly used to assess biodistribution of an ^111^In-labelled radiopharmaceutical in living organisms.^10^ To assess the compatibility of [^111^In][In-**2**]^4+^ with this technique, an exploratory SPECT imaging study in DLD-1 tumour-bearing mice was conducted. SPECT images were acquired from continuous data acquisition 10–70 minutes post intravenous injection (p.i.) with [^111^In][In-**2**]^4+^. As shown in Fig. 5a and S13 in the ESI,† [^111^In][In-**2**]^4+^ accumulated primarily in the liver and bladder with a strong signal in both organs. Biodistribution of [^111^In][In-**2**]^4 +^ 24 h p.i. revealed high liver retention (21.1% injected dose per gram, I.D./g) accompanied by low accumulation in all other regions (<2.4% I.D. per g for all other organs tested, Fig. 5b). Taken together, these results indicate that the liver is the primary organ targeted by [^111^In][In-**2**]^4+^*in vivo*. The total levels of radioactivity recovered from tumours were low (0.09% I.D. per g^–1^, Fig. 5b), indicating poor inherent tumour-targeting properties of the compound.

## Discussion and conclusions

In the area of designing small molecules to bind non-canonical DNA structures, DNA mismatch-interactive compounds have shown encouraging preferential activity towards MMR-deficient colorectal cancers.^44^ While definitive data on the lifetime of mismatches themselves (as opposed to their mutation signatures) in human cells is currently lacking, a genome-wide study in yeast indicated repair of TT mismatches to be one of the least efficient of all mismatch base pairings.^12^ Preferential stabilisation of a TT mismatch is a relatively rare finding for mismatch-interactive compounds described to date.^20^ Related to the chemical design of [**2**]^+^, other molecules that preferentially bind TT mismatches likewise employ a bis-intercalating design. This includes a bis-naphthalene macrocycle “threading” molecule,^45^ a triaminotriazine–acridine conjugate which acts by intercalation of the acridine group along with hydrogen bonding^46^ and a vinyldiaminotriazine–acridine conjugate for the selective alkylation of TT mismatched DNA.^47^ Although our study was limited to a short hairpin and TT mismatch-containing 10-mer duplex, it would be interesting to examine the stabilisation of a greater range of mismatch-containing duplex sequences by these Ru(ii) complexes. Considering X-ray crystal structures of RPC-DNA co-crystals have proven invaluable in understanding binding geometries and specificities,^48^–^51^ X-ray crystallography studies employing [**2**]^+^ and [In-**2**]^4+^ alongside matched and mismatch-containing duplexes would be similarly useful.

Our results indicate that [^111^In][In-**2**]^4+^ generates preferential DNA damage and accompanying decrease in cell survival in MMR-deficient cancer cells by ^111^In decay. To our knowledge, this is the first example of a radiopharmaceutical exhibiting selective activity towards MMR-deficient cancer cells. Evidence of nuclear targeting by [^111^In][In-**2**]^4+^, the ability of [In-**2**]^4+^ to preferentially bind and stabilise TT mismatched DNA along with demonstrable DNA damage foci in MMR-deficient cancer cells are findings that would agree with the notion that [^111^In][In-**2**]^4+^ generates DSB damage at these specific regions. These results are consistent with biological studies demonstrating Auger electrons from ^111^In generate radiotoxic DSB damage when in close proximity to DNA^52^ and computation modelling showing DSBs induced by ^111^In are within 4 nm from the site of decay on the central axis of DNA.^53^ Further biochemical studies on DNA damage generated by [^111^In][In-**2**]^4+^ are required to examine this hypothesis in more detail.

Although the In-chelator H_3_[**2**]^4+^ does bind DNA, it had negligible cytotoxicity and does not generate significant intracellular DNA damage, which is advantageous in its use as a benign carrier of ^111^In to the target DNA molecule. This approach is in contrast to studies examining cytotoxic organic intercalators conjugated to ^99m^Tc.^54^–^56^ As ^99m^Tc is an AE-emitting radiometal with lower efficacy than ^111^In,^9^ in these cases the cytotoxic effects of the organic intercalator dominates and high specific activities of the radiopharmaceutical are required for radiotoxicity. Although these results are encouraging, enhancing the selectivity of H_3_[**2**]^4+^ and [In-**2**]^4+^ towards the mismatch-containing duplex *versus* well-matched sequence is desirable for translational applications. As an example, this could be achieved by chemical modification of the ancillary tpm ligand to decrease affinity for well-matched DNA. The mismatch selectivity of related complexes reported by Boynton, *et al.* demonstrate that optimised targeting of these defects can be accomplished.^27^,^28^

The use of radiolabelled compounds has proven invaluable in drug design as this may provide insight into biodistribution, tumour-targeting, pharmacokinetics and pharmacodynamics in living organisms. Given the recent increase of interest in RPCs as therapeutics,^57^–^59^ this has made understanding these properties highly topical. While the large, hydrophilic [In-**2**]^4+^ is atypical of many RPCs tested in this capacity, the unexpected finding of rapid uptake and retention of [^111^In][In-**2**]^4+^ in the liver combined with low kidney uptake is significant. This is in stark contrast to the majority of organic radiometal chelators^60^ and may be compared directly to biodistribution results for ^111^In[In-DTPA] in mice where no significant retention in any organ was described.^61^ Examples of metal-based chelators for radiometals are exceedingly rare,^62^ however, a structurally-related gadolinium-labelled bis-platinum Pt(NH_3_)_2_Cl–DTPA complex is known to demonstrate high kidney uptake by MRI imaging.^63^ That an evidently different clearance pathway is used by [^111^In][In-**2**]^4+^ is particularly interesting considering the interest in RPCs as alternative therapeutics to platinum drugs and also the fact that clinical use of cisplatin is limited by nephrotoxicity.^64^ We also note with interest that high liver uptake was reported for a hydrophobic mononuclear RPC by the Gasser group,^65^ indicating this may be a general outcome for RPCs. Chemical rerouting of an elimination pathway towards hepatic clearance will aid further design of RPCs as non-platinum chemo/radiotherapeutics, and indicates that hepatotoxicity may be a concern for this class of chemical.

The low intrinsic tumour-targeting of [^111^In][In-**2**]^4+^ and relatively high doses required for radiotoxicity present challenges. While increasing the hydrophobicity of [^111^In][In-**2**]^4+^ with hydrophobic ancillary ligands such as DIP (DIP = 4,7-diphenyl-1,10-phenanthroline) could increase cellular uptake, related research has shown this approach leads to a decrease in nuclear DNA targeting^30^,^66^ and results in potent – but non-specific – cytotoxicity.^65^ Instead, a delivery mechanism may be a more appropriate method to achieve sufficient tumour uptake *in vivo* for the therapeutic potential of this molecule to be assessed in more detail. One attractive option is bioconjugation employing a cleavable linker to a peptide or antibody that targets surface receptors overexpressed by cancers.^67^ Peptide conjugation can substantially improve biodistribution and pharmacokinetics of an administered agent^68^ and numerous design strategies for metallo-drug peptide conjugation have been outlined in a recent review.^69^ Finally, ^111^In radiopharmaceuticals are also compatible with drug-delivery approaches such as liposome-encapsulation of the chelator before subsequent radiometal loading.^70^ A stimuli mediated delivery mechanism then achieves localised drug release and improved tumour uptake.^71^ Future work will explore these concepts.

## Ethical statement

All animal procedures were performed in accordance with the UK Animals (Scientific Procedures) Act 1986 and with local ethical committee approval.

## Conflicts of interest

There are no conflicts to declare.

## Supplementary Material

Supplementary information
