# Supplementary material for: An 111In-labelled bis-ruthenium(ii) dipyridophenazine theranostic complex: mismatch DNA binding and selective radiotoxicity towards MMR-deficient cancer cells
Source: Chem Sci. 2020 Aug 10;11(33):8936–44. doi: 10.1039/d0sc02825h (PMC7989384; doi:10.1039/d0sc02825h)
Supplement: Supplementary file 1 [file SC-011-D0SC02825H-s001.pdf]

Electronic Supplementary Material (ESI) for Chemical Science.
This journal is © The Royal Society of Chemistry 2020

Supplementary information for:

**An $^{111}$In-labelled bis-ruthenium(II) dipyridophenazine theranostic complex: Mismatch DNA binding and selective radiotoxicity towards MMR-deficient cancer cells**

Martin R. Gill,[a,e]* Michael G. Walker,[b] Ole Tietz,[a] Sarah Able,[a] Abirami Lakshminarayanan,[a,d] Rachel Anderson,[a] Rod Chalk,[c] Afaf H. El-Sagheer,[d,f] Tom Brown,[d] Jim A. Thomas[b] and Katherine A. Vallis[a]*

a Oxford Institute for Radiation Oncology, Department of Oncology,

University of Oxford, Oxford

b Department of Chemistry, University of Sheffield, Sheffield, UK

c Structural Genomics Consortium, University of Oxford, Oxford

d Chemistry Research Laboratory, Department of Chemistry, University of Oxford, Oxford OX1 3TA

e Department of Chemistry, Swansea University, Swansea, Wales, UK

f Chemistry Branch, Department of Science and Mathematics, Faculty of Petroleum and Mining Engineering, Suez University, Suez 43721, Egypt

email: m.r.gill@swansea.ac.uk or Katherine.vallis@oncology.ox.ac.uk

## Materials and Methods

### Chemicals and cell lines

All chemicals were purchased from Sigma Aldrich unless otherwise indicated. [$^{111}$In]$InCl_3$ was supplied by Curium Pharma. Antibodies: γH2AX (Millipore), H2AZ, α-tubulin (both Abcam) and β-actin (Sigma). HCT-116, HT-29, DLD-1, HeLa and WI-38 cell lines were purchased from ATCC. DLD-1+Chr2 cells were a kind gift from Dr S. Martin, CRUK Barts centre, UK (original source: Dr Alan Clark, NIEHS). The oligonucleotides to construct the hairpins were purchased from Sigma.

### Synthesis

[Ru(tpm)(dppz)(4-(aminomethyl)pyridine)]$^{2+}$, [**1**]$^{2+}$. [Ru(tpm)(dppz)(Cl)]$^{+}$ was prepared using a method described in Waywell et al..[1] [Ru(tpm)(dppz)(Cl)]$^{+}$ (0.1070g) and $AgNO_3$ (0.0828g) were refluxed in ethanol-water [1:1] for 4 hours in the absence of light. After cooling to room temperature the solution was filtered through celite to remove precipitated AgCl. An excess of 4-(aminomethyl)pyridine (0.2412 g) was added to the solution mixture, which was then refluxed overnight. The volume of the solution was reduced and the product precipitated by addition of aqueous $NH_4PF_6$ which was collected by filtration and washed with cold water and $Et_2O$ and dried *in vacuo*. Mass = 0.0934 g (69 % yield). TOF MS ES+, m/z: 353 [M-2$PF_6$]$^{2+}$, 705 [M-2H]$^{+}$, 851 [M-$PF_6$]$^{+}$. Q-TOF LC/MS: [M-H]: Predicted mass: 704.78. Found: 704.14. $^{1}$H NMR (400 MHz, $CD_3CN$): δH = 9.78 (dd, $J$ = 8.2, 1.2 Hz, 2H), 9.12 (s,1H), 9.12 (dd, $J$ = 5.4, 1.2 Hz, 2H), 8.60 (d, $J$ = 2.9 Hz, 2H), 8.58 (dd, $J$ = 6.6, 3.4 Hz, 2H), 8.37 (d, $J$ = 2.9 Hz, 1H), 8.22 (dd,

*J* = 6.6, 3.4 Hz, 2H), 8.14 (d, *J* = 2.1 Hz, 2H), 8.03 (dd, *J* = 8.2, 5.4 Hz, 2H), 7.45 (dd, *J* = 2.8, 2.4 Hz, 2H), 7.05 (d, *J* = 5.8, 1.5 Hz, 2H), 6.80 (d, *J* = 2.2 Hz, 1H), 6.45 (dd, *J* = 5.7, 1.73 Hz, 2H), 6.20 (dd, *J* = 1.95, 3.0 Hz, 1H), 4.01 (2H).

$H_3$[**2**]$^{4+}$ was prepared by reacting [**1**]$^{2+}$ with cyclic DTPA in a 2:1 molar ratio in dry DMF overnight at 60 °C. The crude product was precipitated by the addition of diethyl ether and collected by centrifugation. The reaction mixture was then analyzed by reverse-phase HPLC. The major peak at 12 min 03 s (Method A) was characterised as $H_3$[**2**]$^{4+}$ by high resolution mass spectrometry. The major peak at 12 min 03 s (HPLC Method A) was collected by reversed-phase HPLC. The solution was pH adjusted to neutral and the acetonitrile removed by rotary evaporation. Addition of $NH_4PF_6$ gave a red-orange precipitate which was collected by centrifugation and washed with diethyl ether. The solid was dried using a Virtis Advantage Plus freeze dryer. NMR: 400 MHz, ($(CD_3)_2CO$): δH 9.61 (br, 4H), 9.38 (br, 6H), 8.54 (br, 4H), 8.43 (br, 4H), 8.18 (br, 6H), 8.10 (br, 4H), 7.65 (br, 4H), 6.95 (br, 4H), 6.65 (br, 4H), 6.34 (br, 6H), 3.87 (dd, *J* = 3.87, 3.84 Hz, 4H), 2.10 (s, 4H), 1.25 (d, *J* = 1.25 Hz, 4H), 1.15 (t, *J* = 1.16 Hz, 4H). Q-TOF LC/MS: [M-3H]+ predicted mass: 1764.79. Found: 1765.37. For "cold" In coordination, $H_3$[**2**]$^{4+}$ (as the chloride salt) was dissolved in water and $InCl_3$ added in a 1:1 ratio. The solution was heated at 90 °C for 1 h. The reaction was monitored by HPLC (Method A) until complete conversion of the 12 min 03 s peak to a new peak at 12 min 47 s. No other HPLC peaks were observed. Q-TOF LC/MS: [M-4H] predicted mass: 1877.59. Found: 1877.23. Due to the hygroscopicity of the dinuclear Ru(II) compounds, we were unable to obtain reliable elemental analyses, even after vacuum drying. All Ru(II) compounds were used as their chloride salts in biological studies.

**Radiolabelling**

A typical radiolabelling reaction involved adding 2 μl of a $H_3$[**2**]$^{4+}$ stock solution (2 mM in $H_2O$) to 20 MBq [$^{111}$In]$InCl_3$ in 0.1 M sodium citrate buffer at pH = 5.5. After 1 h at 20 °C, radiolabelling efficiency was evaluated using instant thin layer chromatography (iTLC, Eckert & Ziegler AR-2000 radio-TLC Imaging Scanner) on glass microfiber chromatography paper impregnated with silicic acid with 0.5 M EDTA (pH = 7.6) as the mobile phase. The Ru(II) complex remains at the origin while free (uncoordinated) [$^{111}$In]$In^{3+}$ migrates with the mobile phase. Radiolabelling efficiencies of >95% were required for all biological experiments.

**HPLC**

Reverse-phase HPLC was carried out on an Agilent 1260 Infinity II system fitted with quaternary pump module, vial sampler, multicolumn thermostat, diode array detector and analytical fraction collector (Agilent Technologies, USA). HPLC columns were kept at 30 °C, chromatograms were collected at 210, 230, 250, 254, 280 nm and 440 - 550 nm (at 5 nm intervals) using a deuterium lamp. Radioactivity peaks were detected using a radio-HPLC detector (LabLogic). HPLC purification and quality control samples were run using mobile phases A = $H_2O$ [HPLC grade] (+ 0.1% trifluoroacetic acid, TFA, v/v) and B = $CH_3CN$ [HPLC grade] (+ 0.1% TFA, v/v) and the following two methods:

Method A: Agilent Prep C18 (100 Å, 5.0 μm, 10 x 250 mm) preparative column. Flow rate 3 ml min$^{-1}$. Gradient of 80 % A: 20 % B at t = 0 min rising to 40 % A: 60 % B at t = 30 mins.

Method B: Waters XBridge C18 (130 Å, 3.5 μm, 4.6 x 150 mm) analytical column. Flow rate 1 ml min$^{-1}$. Gradient of 80 % A: 20 % B at t = 0 min rising to 20 % A: 80 % B at t = 20 mins.

**High resolution mass spectrometry**

Samples were diluted to a final concentration of 1 μM in 0.1 % formic acid. 50 μl was injected on to a 1290 UPLC coupled to a 6530 QTOF mass spectrometer fitted with a 2.1 mm x 12.5 mm Zorbax 5 μm 300SB-C3 guard column (Agilent Technologies, Santa Clara, USA). Solvent A was 0.1 % formic acid in HPLC grade water; solvent B was 0.1 % formic acid in LC-MS grade methanol (Fisher Scientific, Loughborough, UK). Initial conditions were 10 % B at 1 ml/min. A methanol gradient was developed from 10 % to 80 % over 34 seconds, followed by isocratic at 95 % B for 41 seconds, followed by 15 seconds re-equilibration at 10 % B. The mass spectrometer was operated in positive ion, 2 GHz detector mode. Source parameters were drying gas 350 °C, flow 12 L/min, nebulizer 60 psi, capillary 4000 V. Fragmentor was 250 V, collision energy 0 V and data acquired from 100-3200 m/z. Data analysis was performed using Masshunter Qualitative Analysis B0.7 proprietary software and deconvolution performed using the resolved isotope method.

**Oligonucleotides synthesis and purification**

Oligonucleotides were synthesized on an Applied Biosystems 394 automated DNA/RNA synthesizer using a standard 1.0 $\mu$mole phosphoramidite cycle of acid-catalyzed detritylation, coupling, capping, and iodine oxidation. Stepwise coupling efficiencies and overall yields were determined by the automated trityl cation conductivity monitoring facility and was >98.0 %. Standard DNA phosphoramidites and additional reagents were purchased from Link Technologies Ltd, Sigma-Aldrich, Glen research and Applied Biosystems Ltd. All β-cyanoethyl phosphoramidite monomers were dissolved in anhydrous acetonitrile to a concentration of 0.1 M immediately prior to use with coupling time of 50 s. Cleavage and deprotection were achieved by exposure to concentrated aqueous ammonia solution for 60 min at room temperature followed by heating in a sealed tube for 5 h at 55 °C. Purification was carried out by reversed-phase HPLC on a Gilson system using a Brownlee Aquapore column (C8, 8 mm x 250 mm, 300 Å pore) with a gradient of acetonitrile in triethylammonium bicarbonate (TEAB) increasing from 0 % to 50 % buffer B over 30 min with a flow rate of 4 mL/min (buffer A: 0.1 M triethylammonium bicarbonate, pH 7.0, buffer B: 0.1 M triethylammonium bicarbonate, pH 7.0 with 50 % acetonitrile). Elution was monitored by ultraviolet absorption at 298 nm. After HPLC purification, oligonucleotides were freeze dried then dissolved in water without the need for desalting. All purified oligonucleotides were characterised by electrospray mass spectrometry. Mass spectra of oligonucleotides were recorded using a XEVO G2-QTOF MS instrument in ES- mode. Data were processed using MaxEnt and in all cases confirmed the integrity of the sequences.

### DNA binding

Calf thymus DNA was dissolved in aqueous Tris-buffer (25 mM NaCl, 5 mM Tris, pH = 7) and broken into an average of 150-200 base pair fragments by sonication (2 × 15 mins). Purity was determined by UV-vis spectroscopy, with $A_{260}/A_{280} > 1.8$ indicating a protein-free sample. Luminescent titrations were carried out with the addition of an aqueous solution of concentrated DNA to 30 μM of each Ru(II) complex. After each addition of DNA, the solution was mixed by pipette and allowed to equilibrate for 5 min. Luminescence spectra were recorded on a Tecan Infinite 200 PRO Microplate Reader ($\lambda_{ex}$ = 405 nm, $\lambda_{em}$ = 500-800 nm). DNA was added until the MLCT emission reached a maximum (typical DNA concentration at saturation = 80-90 μM). The AUC (area under curve) for each luminescence spectrum were used to construct nonlinear Scatchard plots (r/Cf versus r, an example within Fig S7b) and fit to the McGhee-von Hippel model,[2] in which neither the site size nor binding constant were defined, to determine $K_b$ and n. Hairpin DNA and each 10-mer DNA duplex were dissolved in 10 mM phosphate buffer, 200 mM NaCl at pH 7.0. A 3 μM concentration of each was then added to 30 μM of each Ru(II) complex in 10 mM phosphate buffer, 200 mM NaCl at pH 7.0 and the fluorescence spectrum recorded on a Tecan Infinite 200 PRO Microplate Reader ($\lambda_{ex}$ = 405 nm, $\lambda_{em}$ = 500-800 nm). The emission intensity was determined as the AUC.

### Ultraviolet DNA melting studies

UV DNA melting was monitored on Cary 4000 Scan UV-Visible Spectrophotometer (Varian) using an adapted method of a recent publication.[3] A 3 μM concentration of each oligonucleotide +/- 30 μM of each Ru(II) complex in 10 mM phosphate buffer, 200 mM NaCl at pH 7.0 was employed. The samples were initially denatured by heating to 85 °C at 10 °C $min^{-1}$, cooled to 20 °C at 1 °C $min^{-1}$ and then heated to 85 °C at 1 °C $min^{-1}$. Spectra were recorded at 260 nm. Eight successive melting curves were measured and $T_m$ values were calculated from their derivatives using Cary Win UV Thermal application Software. $\Delta T_m = T_m$ (with compound) - $T_m$ (without compound).

### Cell culture

HCT-116, HT-29, WI-38 and HeLa cells were cultured in DMEM supplemented with 10% FBS and penicillin/streptomycin. DLD-1 and DLD-1+Chr2 cells were cultured in RPMI supplemented with 10% FBS and penicillin/streptomycin. DLD1+Chr2 cells were maintained under selective pressure of 400 µg/mL geneticin (G418 sulfate, Roche). Cell lines were maintained at 37 °C in an atmosphere of 5% $CO_2$ and sub-cultured by Trypsin. Cell lines were used at passage numbers 40 or lower and routinely confirmed to be mycoplasma-free.

### Subcellular distribution

Non-radioactive compounds: HCT-116 cells were grown in 6 well plates, treated with Ru(II) compound for 24 h, washed with cold PBS (2 × 2 mL) before washing with acidified PBS (pH 2.5) to remove the surface-bound fraction. 0.4 mL EZ lysis buffer was added, cells were detached by scraping, collected

into eppendorf tubes, vortexed briefly and left for 5 minutes on ice. Samples were centrifuged (500 g, 5 min) and the supernatant (cytosol fraction) aspirated. The pellet (nuclear fraction) was re-suspended in 200 µl RIPA buffer. Successful fractionation of the two subcellular compartments was verified by immunoblotting using anti-α-tubulin (Sigma) and anti-histone H2AZ (Abcam) for cytosol and nuclear fractions, respectively. Protein content of each fraction was determined by BCA assay and ruthenium content determined by ICP-MS analysis, as described in detail in a recent publication.[4]

Radioactive compounds: The adherence of $[^{111}In]In^{3+}$ to the negatively-charged tissue culture plastic well plates gave a false signal by the method above. To counter this, $1x10^6$ cells in eppendorfs were treated with 1 MBq/ml $[^{111}In][In$-**2**$]^{4+}$ for 2 h with gentle shaking. Cells were collected by centrifugation (2000 rpm, 5 min) before processing as described above. Protein content of each fraction was determined by BCA assay and radioactivity was measured using a WIZARD-2 Automatic Gamma Counter.

**Confocal microscopy**

HCT-116 cells were seeded in ibidi 35 mm µ-dishes (Thistle Scientific) 24 h before treatment with [**1**]$^{2+}$ or $H_3$[**2**]$^{4+}$ (100 µM, 4 h in serum-free media). Samples were visualised using a Zeiss LSM 780 inverted confocal microscope and an EC Plan-Neofluar 40×/1.30 Oil objective. Excitation wavelength = 405 nm, emission wavelength = 600-700 nm.

**MTT assay**

Cancer cell lines were seeded in 96 well plates at 10,000 cells/well and allowed to adhere for 24 h before treatment. Cell lines were treated with a concentration gradient of each compound in triplicate. After incubation, 0.5 mg/ml MTT (thiazolyl blue tetrazolium bromide) dissolved in serum-free medium was added for 60 minutes and the formazan product eluted using acidified isopropanol. The absorbance at 540 nm was quantified by plate reader (reference wavelength 650 nm). The metabolic activity of the cell population was determined as a percentage of a negative (solvent) control. WI-38 cells were seeded in 24 well plates at 10,000 cells/well and allowed to adhere for 24 h. Cells were treated with 0-10 MBq/ml $[^{111}In][In$-**2**$]^{4+}$ or $[^{111}In]InCl_3$ (as citrate) for 24 h. This was removed and the cells washed with PBS. Fresh media was added and the cell viability determined after 72 h by MTT assay.

**Clonogenic survival assay**

Radioactive compounds: Cells were grown in 48 well plates and treated with 0-10 MBq/ml $[^{111}In][In$-**2**$]^{4+}$ or $[^{111}In]InCl_3$ (as citrate) for 24 h. For radiosensitization experiments, HCT-116 cells were treated with non-radioactive [**1**]$^{2+}$, $H_3$[**2**]$^{4+}$ or [In-**2**]$^{4+}$ for 24 h before exposure to 0-6 Gy $^{137}Cs$-γ-rays. Cells were washed with PBS and collected using Trypsin. Samples were counted and cells re-seeded in 6 well plates at 1000-5000 cells/well (exact number optimised for each cell line and condition to ensure adequate colonies for counting).

External beam: DLD-1 or DLD-1+Chr2 cells were seeded in 6 well plates at 1500-48000 cells/well (depending on dose to be received) and allowed to adhere for 4 h. Cells were then exposed to 0-8 Gy $^{137}$Cs-$\gamma$-rays.

In all cases, colonies were grown for >7 days, washed in PBS, and stained with 1 % methylene blue (Alfa Aesar) in 50% methanol (Thermo Fisher Scientific). Colonies containing 50 cells or greater were counted using a Gelcount instrument and accompanying software (Oxford Optronix). Plating efficiencies were determined for each treatment condition and normalised to an untreated control to provide the surviving fraction (S. F.).

**Western blotting and immunofluorescence**

Non-radioactive compounds: HCT-116 cells were seeded in 6 well plates at $2x10^5$ cells/well, left for 24 h and then treated with each Ru(II) compound for 24 h (50 $\mu$M). After incubation, cells were washed with PBS and detached by scraping. Radioactive compounds: $8x10^5$ cells were treated with 10 MBq/ml [$^{111}$In][In-**2**]$^{4+}$ for 6 h in eppendorfs with gentle shaking. 10 MBq/ml [$^{111}$In]$InCl_3$ (as citrate) or unlabelled $H_3$[**2**]$^{4+}$ treatment groups were employed in parallel. After incubation, cells were collected by centrifugation (2000 rpm, 5 min) and washed with PBS. In both cases, cell lysates were prepared and western blotting performed precisely as described in a recent publication using WesternSurePREMIUM (Li-Cor) chemiluminescent substrate and a LiCor C-Digit Blot Scanner.[5] Antibody concentrations: $\gamma$H2AX (1/1000), $\beta$-actin (1/5000), $\alpha$-tubulin (1/5000), H2AZ (1/1000). HRP-conjugated anti-mouse or anti-rabbit secondary antibodies were used at 1/5000 dilutions. All antibodies were diluted in 5% BSA in TBST. For immunofluorescence, HCT-116 cells were grown in Ibidi $\mu$dishes, treated with 10 MBq/ml [$^{111}$In]$InCl_3$ (as citrate) or [$^{111}$In][In-**2**]$^{4+}$ for 6 h before fixation and immunofluorescent staining for $\gamma$H2AX and nuclear staining using DAPI exactly as described within a recent publication.[5] Cells exposed to 3 Gy $^{137}$Cs-$\gamma$-rays, fixed and stained 1 h after exposure, were used as a positive control.

**Animal procedures and SPECT imaging**

All animal procedures were performed in accordance with the UK Animals (Scientific Procedures) Act 1986 and with local ethical committee approval. DLD-1 tumours were established by subcutaneous injection of $2 \times 10^6$ cells suspended in 200 µL 1:1 RPMI:Matrigel into the right dorsal flank of female BALB/c Nu/Nu mice (n=3). SPECT imaging was performed using a VECTor[4] imaging system (MILabs) using a rat collimator with 1.8 mm pinholes. Continuous SPECT data were acquired for 10-70 minutes after intravenous administration of [$^{111}$In][In-**2**]$^{4+}$ (7-9 MBq) followed by a CT image on the same system. Image acquisition and processing was performed as described in detail elsewhere using the PMod software package.[6] Mice were kept under anaesthesia by inhalation of 2% isofluorane in air and maintained at 37 °C during the imaging session. Mice were revived and 24 h after the SPECT imaging session they were euthanised by cervical dislocation. Selected organs, tissues and blood were removed, and the percentage of the injected dose per gram (% ID/g) of each sample was calculated. No adverse effects of the compound were observed.

**Supporting Information Tables**

Table S1 DNA binding constants ($K_b$) for Ru(II) complexes with calf thymus DNA, as determined from luminescence titration data. n = binding site size (in base pairs).

| Compound | $K_b$ ($M^{-1}$) | n |
|---|---|---|
| [**1**]$^{2+}$ | $3.8 \pm 0.5 \times 10^5$ | $1.3 \pm 0.05$ |
| $H_3$[**2**]$^{4+}$ | $2.4 \pm 0.2 \times 10^5$ | $1.7 \pm 0.05$ |
| [In-**2**]$^{4+}$ | $1.1 \pm 0.2 \times 10^6$ | $1.8 \pm 0.03$ |

Table S2 Description of cell lines used in this study. [a]Data from ref 7 in the SI. [b]Determined by clonogenic survival assay

| Cell line | Origin | MMR status | Mutation frequency (mutations per Mb)[a] | S.F. 3 Gy Cs $\gamma$-rays[b] |
|---|---|---|---|---|
| HCT-116 | Colorectal | MMR- (-MLH1) | 95.68 | 0.27 ± 0.03 |
| DLD-1 | Colorectal | MMR- (-MSH6) | 260.69 | 0.52 ± 0.06 |
| HT29 | Colorectal | MMR+ | 17.03 | 0.40 ± 0.11 |
| HeLa | Cervical | MMR+ | U/K | 0.53 ± 0.06 |

Table S3 $IC_{50}$ values (μM, 72 h incubation) of non-radioactive Ru(II) compounds and cisplatin towards HCT-116, DLD-1, HT-29 (all colorectal cancer) or HeLa cervical cancer cell lines, as determined by MTT assay.

| Compound | HCT-116 | DLD-1 | HT-29 | HeLa |
|---|---|---|---|---|
| $[\mathbf{1}]^{2+}$ | >100 | >100 | >100 | >100 |
| $H_3[\mathbf{2}]^{4+}$ | >50 | >50 | >50 | >50 |
| $[In\text{-}\mathbf{2}]^{4+}$ | 34 | >50 | >50 | 32 |
| Cisplatin | 6.6 | 18 | 38.9 | 0.5 |

Table S4 Sensitivity of HCT-116 cells to γ-rays after treatment with Ru(II) compounds (24 h). Extrapolated dose resulting in surviving fraction (S.F.) of 0.1 shown. DMF = dose modification factor = S.F. [without compound]/ S.F. [with compound]. See Figure S11.

| Compound | Gy (S.F.= 0.1) | DMF (S.F.= 0.1) |
|---|---|---|
| Blank | 3.95 | - |
| $[\mathbf{1}]^{2+}$ | 3.75 | 1.05 |
| $H_3[\mathbf{2}]^{4+}$ | 3.43 | 1.15 |
| $[In\text{-}\mathbf{2}]^{4+}$ | 2.9 | 1.36 |

## Supporting Information Figures

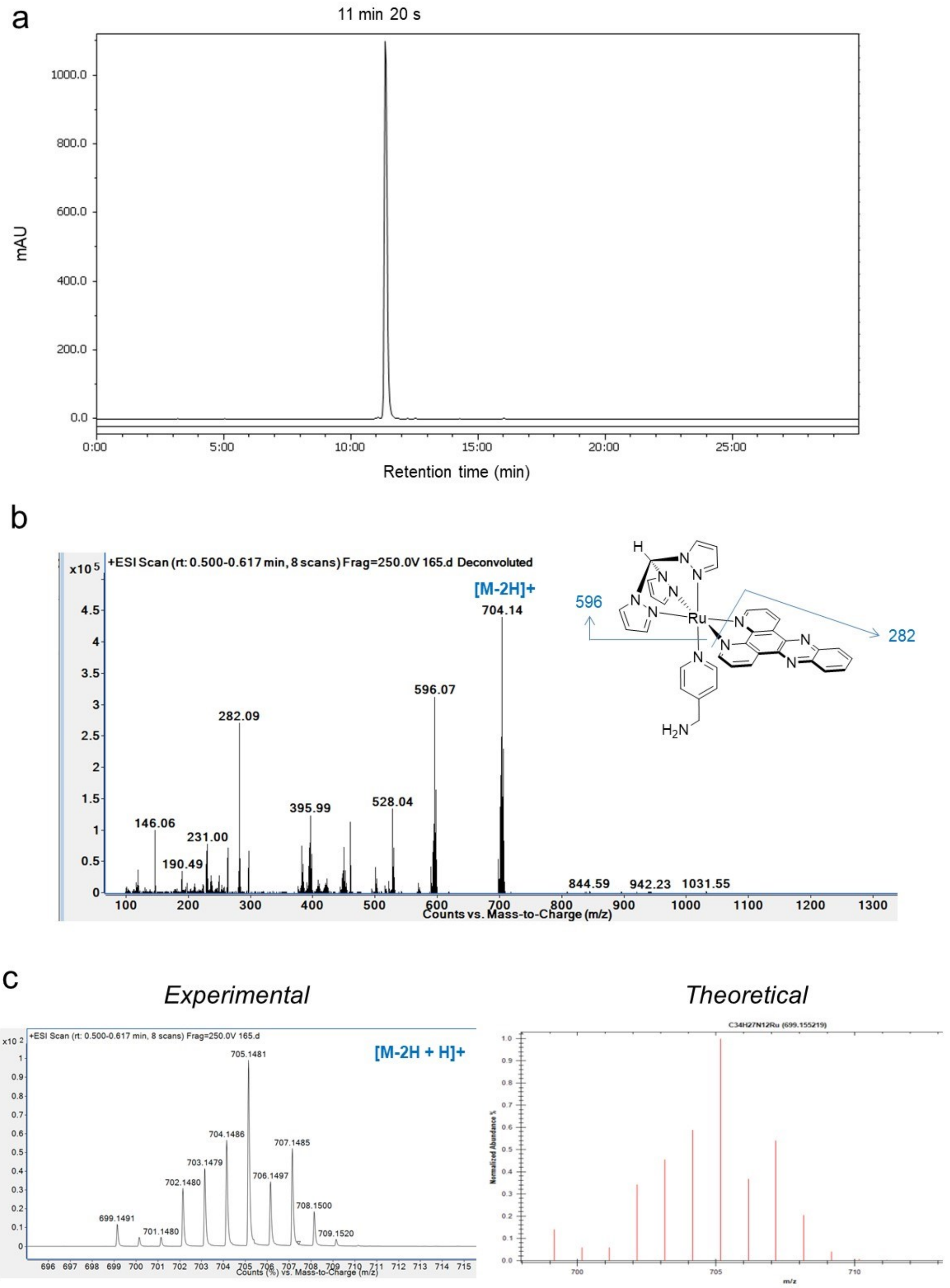


Figure S1 (a) HPLC chromatogram of [**1**]$^{2+}$. HPLC Method A. (b) Deconvoluted Q-LC/MS spectrum of [**1**]$^{2+}$ showing fragmentation peaks. (c) Experimental (left) and theoretical (right) isotope ratio of [M-2H+H]+ peak from (b).

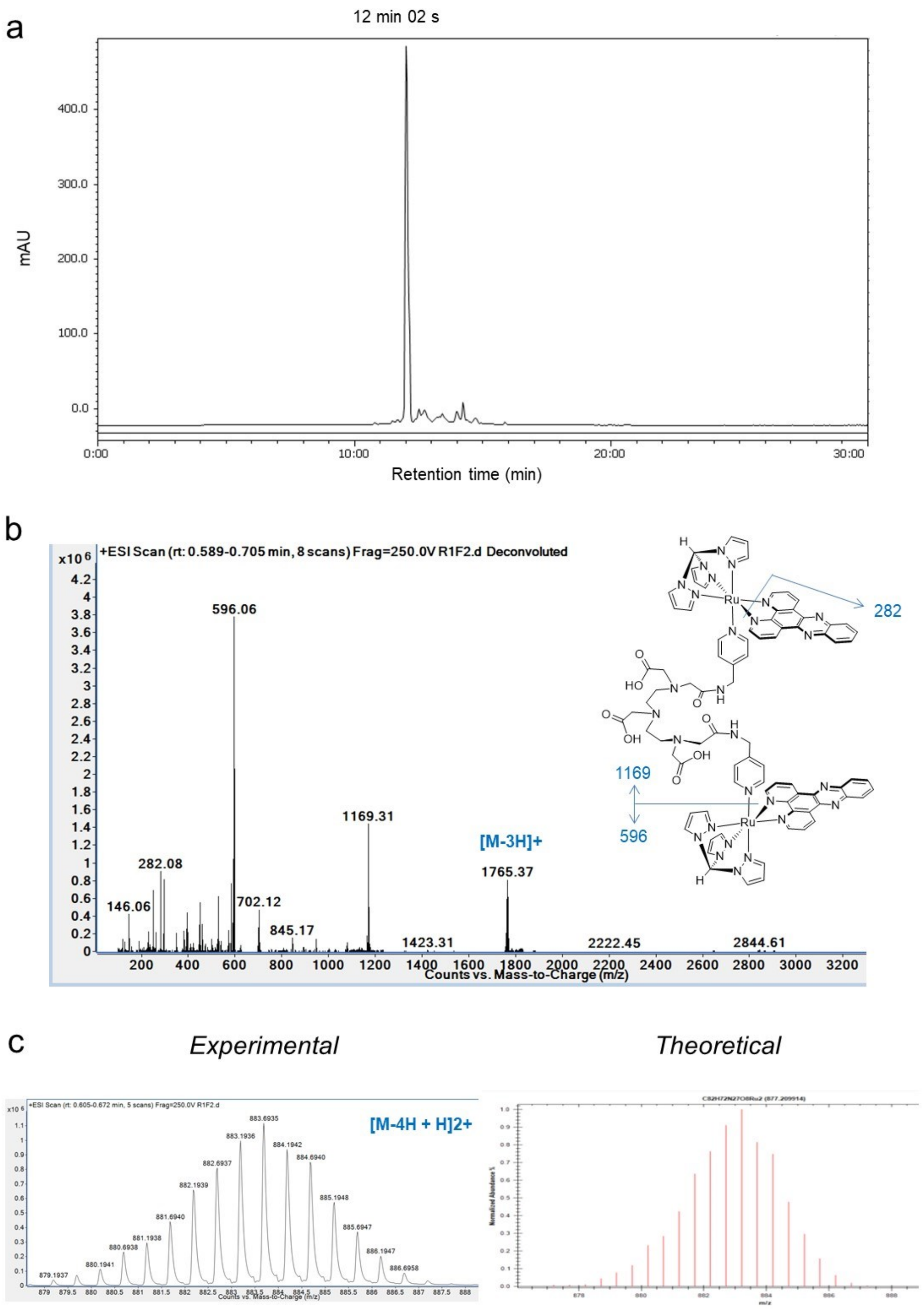


Figure S2 (a) HPLC chromatogram of $H_3[\mathbf{2}]^{4+}$. HPLC Method A. (b) Deconvoluted Q-LC/MS spectrum of $H_3[\mathbf{2}]^{4+}$ showing fragmentation peaks. (c) Experimental (left) and theoretical (right) isotope ratio of [M-4H+H]2+ peak from (b).

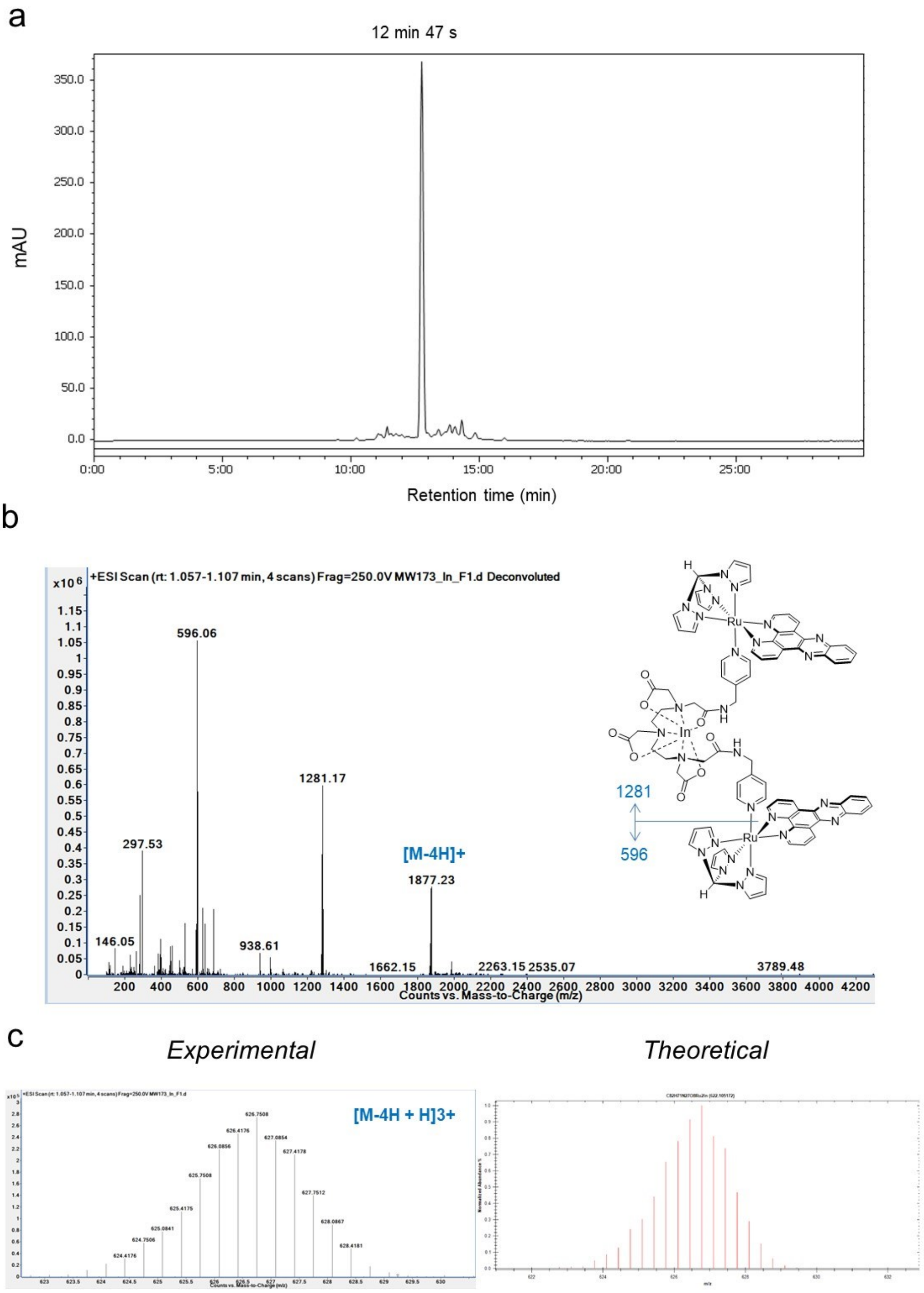


Figure S3 a) HPLC chromatogram of [In-**2**]$^{4+}$. HPLC Method A. (b) Deconvoluted Q-LC/MS spectrum of [In-**2**]$^{4+}$ showing fragmentation peaks. (c) Experimental (left) and theoretical (right) isotope ratio of [M-4H+H]3+ peak from (b).

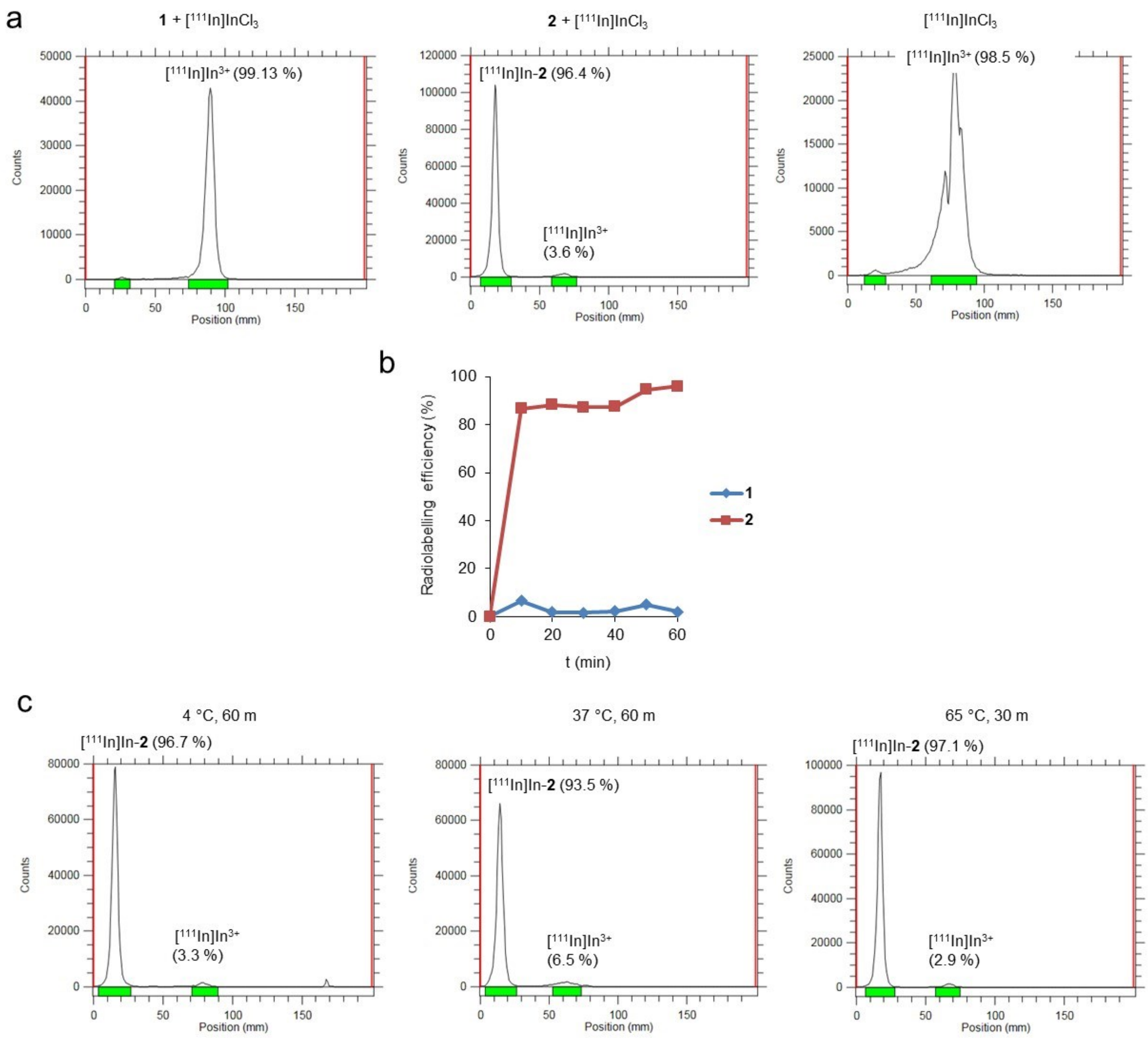


Figure S4 (a) Representative instant thin layer chromatograms (iTLC) of $[\mathbf{1}]^{2+}$ or $H_3[\mathbf{2}]^{4+}$ after addition of $[^{111}In]In^{3+}$ (60 m, 20 °C). EDTA (0.5 M, pH = 7.6) was employed as the mobile phase and $[^{111}In]In^{3+}$ was added as $[^{111}In]InCl_3$ in citrate buffer. (b) Quantification of $[^{111}In]$In-binding from iTLC results. (c) Impact of temperature upon formation of $[^{111}In][In\text{-}\mathbf{2}]^{4+}$ from $H_3[\mathbf{2}]^{4+}$ and $[^{111}In]In^{3+}$.

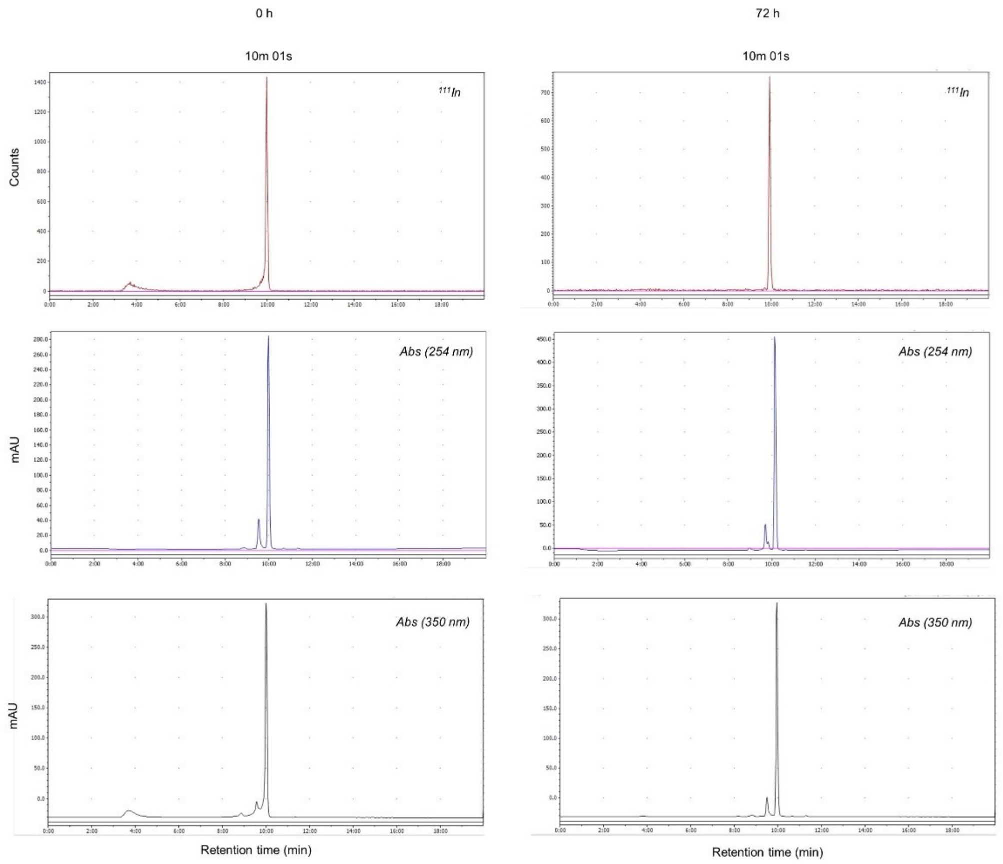


Figure S5 HPLC chromatograms of co-injection of [$^{111}$In][In-**2**]$^{4+}$ and [In-**2**]$^{4+}$ showing radioactivity (top row) and absorbance (middle and bottom rows). 0 h and 72 h after radiolabelling (storage conditions: 4 °C). HPLC Method B.

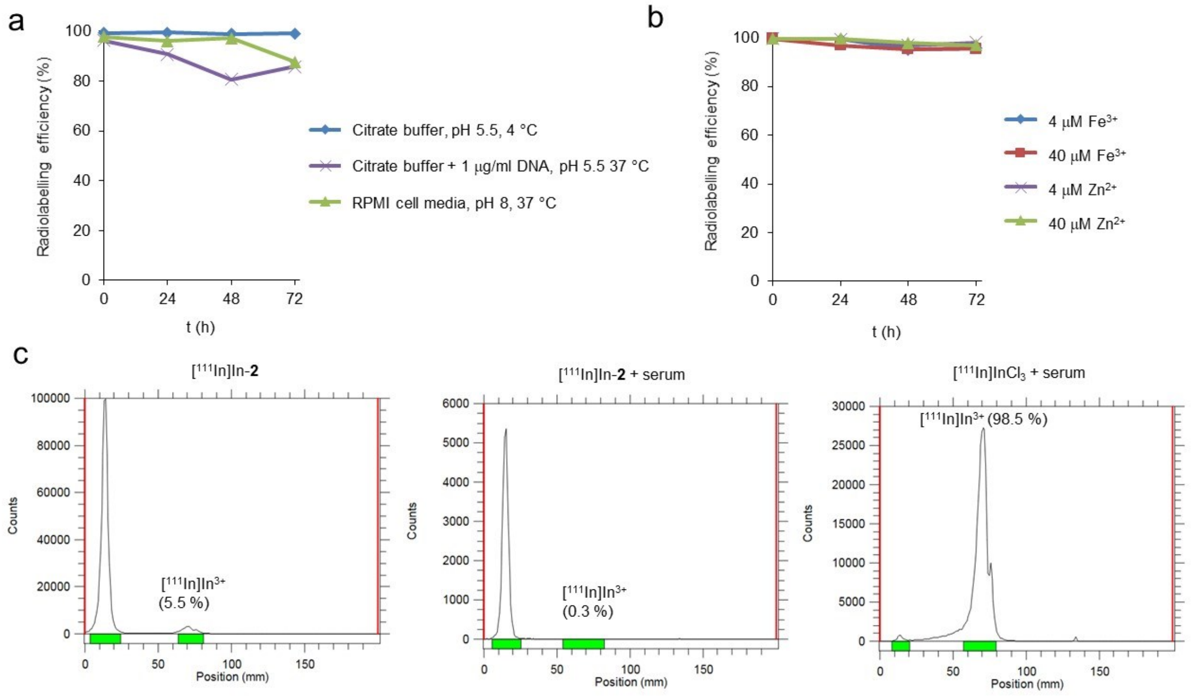


Figure S6 (a) Quantification of [111In]In-binding to $H_3[\mathbf{2}]^{4+}$ from iTLC results in the presence of DNA or in RPMI cell media. (b) Impact of competing metal ions on [111In]In-binding to $H_3[\mathbf{2}]^{4+}$. (c) iTLC of [111In][In-**2**]$^{4+}$ before and after addition of mouse serum (24 h incubation, 37 °C). Results for [111In]$In^{3+}$ with the addition of serum included (right).

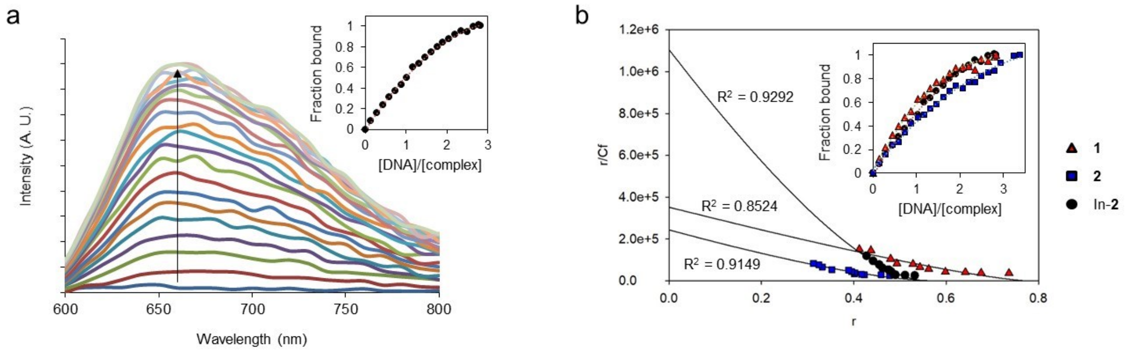


Figure S7 (a) Example of MLCT luminescence titration of Ru(II) complex ([In-**2**]$^{4+}$, 30 μM) with the addition of calf-thymus DNA ($\lambda_{ex}$ = 405 nm). Derived binding curve in inset. (b) Scatchard plots from luminescent titrations. Binding curves in inset.

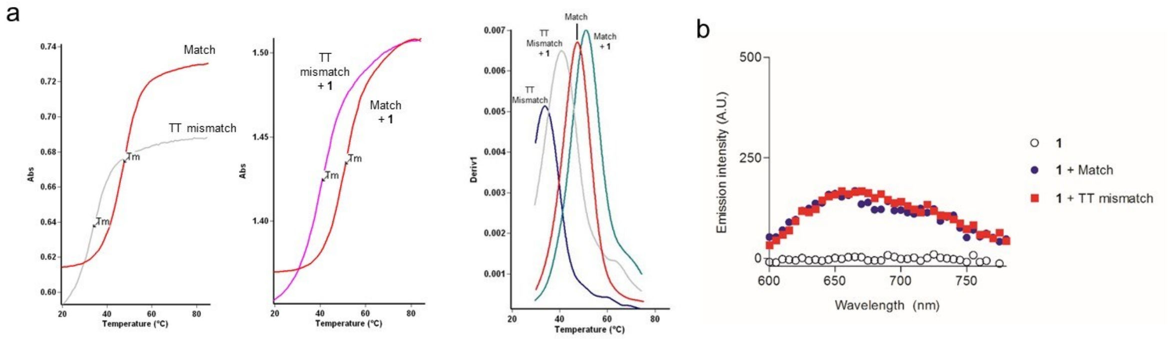


Figure S8 (a) Example of DNA melting curves in absence and presence of Ru(II) complex ([**1**]$^{2+}$ shown, 10:1 complex:DNA ratio). UV melting curves were recorded at 260 nm. (b) MLCT emission of [**1**]$^{2+}$ with the addition of well-matched or mismatch containing duplex.

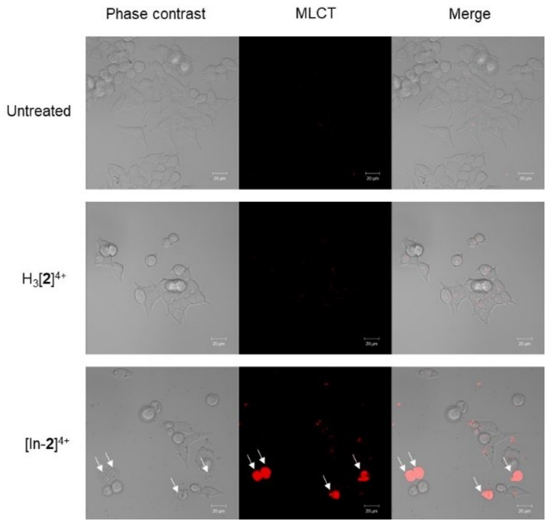


Figure S9 Confocal laser scanning micrograph of HCT-116 cells treated with $H_3$[**2**]$^{4+}$ or [In-**2**]$^{4+}$ (100 µM, 2 h). Arrows: morphologically non-viable cells in [In-**2**]$^{4+}$ treatment group with intense MLCT emission.

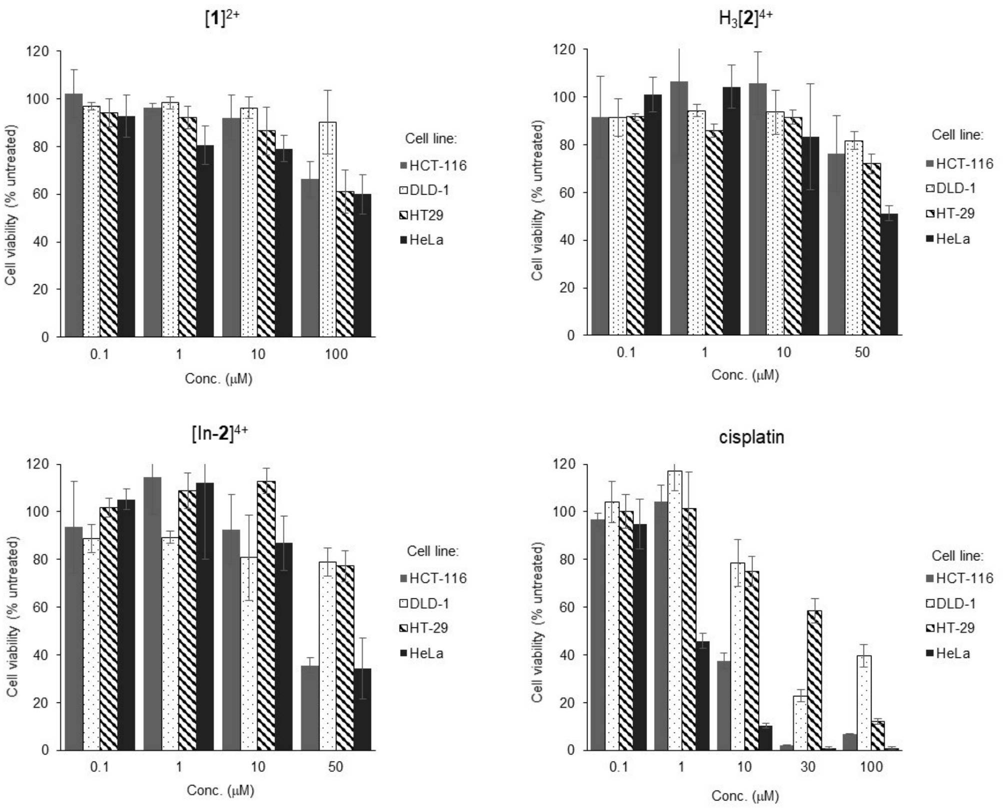


Figure S10 Impact of Ru(II) complexes or cisplatin on viability of HCT-116, DLD-1, HT-29 or HeLa cancer cells, as determined by MTT assay (72 h incubation time). Mean of triplicates +/- S.D..

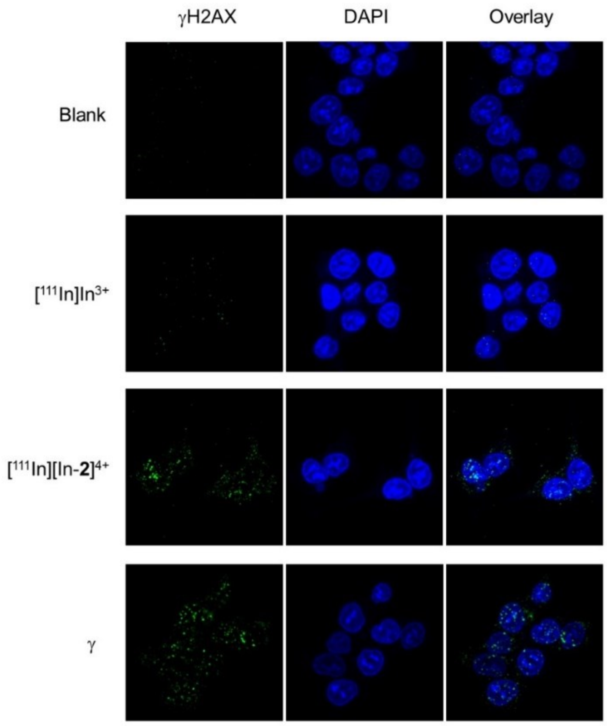


Figure S11 CLSM images of HCT-116 cells treated with [$^{111}$In]$In^{3+}$ or [$^{111}$In][In-**2**]$^{4+}$ (10 MBq/ml, 6 h) or $^{137}$Cs-γ-rays (3 Gy) followed by immunofluorescence staining for γH2AX (green). DNA was stained with DAPI (blue).

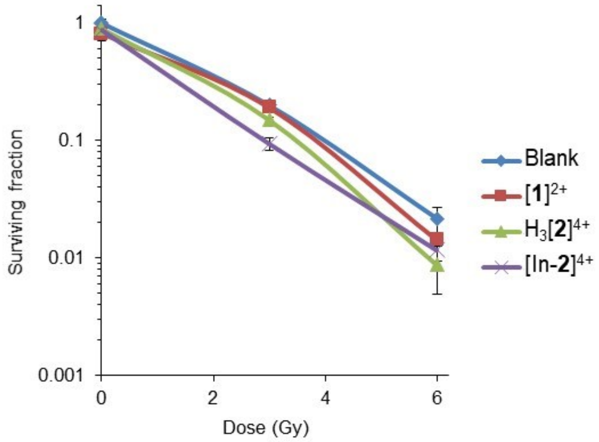


Figure S12 Clonogenic survival of HCT-116 cells pre-treated with Ru(II) compounds (50 μM, 24 h) before irradiation with 0–6 Gy $^{137}$Cs-γ-rays. Mean of triplicates +/- S.D.

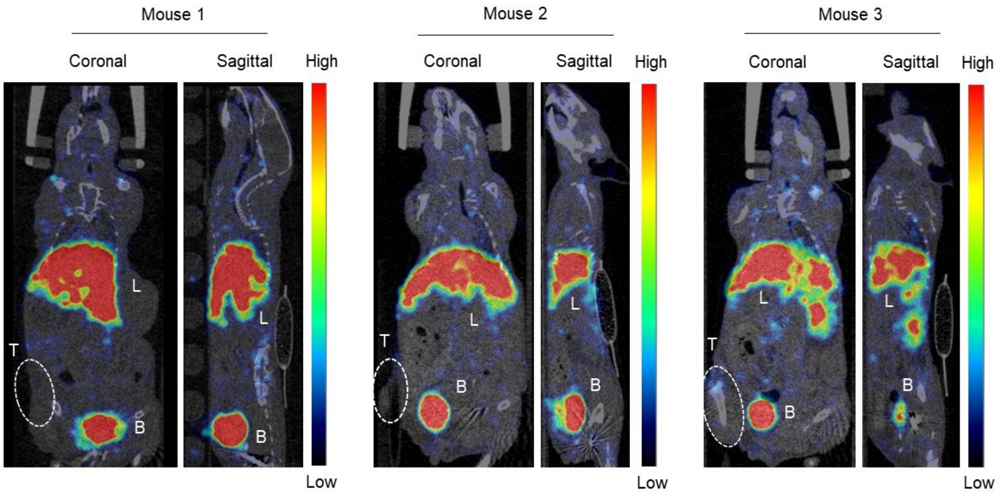


Figure S13 SPECT/CT images of mice constructed from images taken 10-70 min after intravenous injection of [$^{111}$In][In-**2**]$^{4+}$. L = liver, B = bladder, T = tumour.

**Supporting Information References**

1 P. Waywell, V. Gonzalez, M. R. Gill, H. Adams, A. J. H. Meijer, M. P. Williamson and J. A. Thomas, *Chem. - A Eur. J.*, 2010, **16**, 2407–2417.

2 J. D. McGhee and P. H. von Hippel, *J. Mol. Biol.*, 1974, **86**, 469–489.

3 A. H. El-Sagheer and T. Brown, *Chem. Sci.*, 2014, **5**, 253–259.

4 M. R. Gill, S. N. Harun, S. Halder, R. A. Boghozian, K. Ramadan, H. Ahmad and K. A. Vallis, *Sci. Rep.*, 2016, **6**, 31973.

5 M. R. Gill, P. J. Jarman, S. Halder, M. G. Walker, H. K. Saeed, J. A. Thomas, C. Smythe, K. Ramadan and K. A. Vallis, *Chem. Sci.*, 2018, **9**, 841–849.

6 J. C. Knight, J. Baguna Torres, R. Goldin, M. Mosley, G. M. Dias, L. Contreras Bravo, V. Kersemans, P. D. Allen, S. Mukherjee, S. C. Smart and B. Cornelissen, *J. Nucl. Med.*, 2019, **61**, 1006–1013.

7 D. Mouradov, C. Sloggett, R. N. Jorissen, C. G. Love, S. Li, A. W. Burgess, D. Arango, R. L. Strausberg, D. Buchanan, S. Wormald, L. O'Connor, J. L. Wilding, D. Bicknell, I. P. M. Tomlinson, W. F. Bodmer, J. M. Mariadason and O. M. Sieber, *Cancer Res.*, 2014, **74**, 3238–3247.
